# Systemic and skin-limited delayed-type drug hypersensitivity reactions associate with distinct resident and recruited T cell subsets

**DOI:** 10.1172/JCI178253

**Published:** 2024-07-23

**Authors:** Pranali N. Shah, George A. Romar, Artür Manukyan, Wei-Che Ko, Pei-Chen Hsieh, Gustavo A. Velasquez, Elisa M. Schunkert, Xiaopeng Fu, Indira Guleria, Roderick T. Bronson, Kevin Wei, Abigail H. Waldman, Frank R. Vleugels, Marilyn G. Liang, Anita Giobbie-Hurder, Arash Mostaghimi, Birgitta A.R. Schmidt, Victor Barrera, Ruth K. Foreman, Manuel Garber, Sherrie J. Divito

**Affiliations:** 1 Department of Dermatology, Brigham and Women’s Hospital (BWH), Harvard Medical School, Boston, Massachusetts, USA.; 2 Bioinformatics Core,; 3 Bioinformatics and Integrative Biology Program, and; 4 Department of Dermatology, University of Massachusetts Medical School, Worcester, Massachusetts, USA.; 5 Department of Pathology, BWH, Harvard Medical School, Boston, Massachusetts, USA.; 6 Department of Pathology, Beth Israel Deaconess Medical Center, and; 7 Department of Microbiology and Immunobiology, Harvard Medical School, Boston, Massachusetts, USA.; 8 Division of Rheumatology, Inflammation, and Immunity, BWH and Harvard Medical School, Boston, Massachusetts, USA.; 9 Department of Dermatology, Boston Children’s Hospital (BCH), Harvard Medical School, Boston, Massachusetts, USA.; 10 Department of Data Science, Dana Farber Cancer Institute, Boston, Massachusetts, USA.; 11 Department of Pathology, BCH, Boston, Massachusetts, USA.; 12 Bioinformatics Core, Department of Biostatistics, Harvard T.H. Chan School of Public Health, Boston, Massachusetts, USA.; 13 Department of Pathology, Massachusetts General Hospital, Boston, Massachusetts, USA.

**Keywords:** Dermatology, Immunology, Allergy, Skin, T cells

## Abstract

Delayed-type drug hypersensitivity reactions are major causes of morbidity and mortality. The origin, phenotype, and function of pathogenic T cells across the spectrum of severity require investigation. We leveraged recent technical advancements to study skin-resident memory T cells (TRMs) versus recruited T cell subsets in the pathogenesis of severe systemic forms of disease, Stevens-Johnson syndrome/toxic epidermal necrolysis (SJS/TEN) and drug reaction with eosinophilia and systemic symptoms (DRESS), and skin-limited disease, morbilliform drug eruption (MDE). Microscopy, bulk transcriptional profiling, and single-cell RNA-sequencing (scRNA-Seq) plus cellular indexing of transcriptomes and epitopes by sequencing (CITE-Seq) plus T cell receptor sequencing (TCR-Seq) supported clonal expansion and recruitment of cytotoxic CD8^+^ T cells from circulation into skin along with expanded and nonexpanded cytotoxic CD8^+^ skin TRM in SJS/TEN. Comparatively, MDE displayed a cytotoxic T cell profile in skin without appreciable expansion and recruitment of cytotoxic CD8^+^ T cells from circulation, implicating TRMs as potential protagonists in skin-limited disease. Mechanistic interrogation in patients unable to recruit T cells from circulation into skin and in a parallel mouse model supported that skin TRMs were sufficient to mediate MDE. Concomitantly, SJS/TEN displayed a reduced Treg signature compared with MDE. DRESS demonstrated recruitment of cytotoxic CD8^+^ T cells into skin as in SJS/TEN, yet a pro-Treg signature as in MDE. These findings have important implications for fundamental skin immunology and clinical care.

## Abstract 2

Skin-resident memory T cells may mediate skin-limited morbilliform drug eruptions, while clonal expansion and recruitment of circulating cytotoxic CD8^+^ T cells underlie life-threatening skin reactions.

## Introduction

Delayed-type drug hypersensitivity reactions (dtDHRs) are a major cause of morbidity and mortality, with considerable cost to healthcare systems (1–5). Skin is the most commonly affected organ. Severity ranges from a mild skin-limited reaction (morbilliform drug eruption [MDE]) to life-threatening severe cutaneous adverse reactions (SCARs) with skin and systemic involvement. The most severe forms are Stevens-Johnson syndrome/toxic epidermal necrolysis (SJS/TEN), notable for blistering and sloughing of skin and mucosal tissues requiring burn unit level care, and drug reaction with eosinophilia and systemic symptoms (DRESS), notable for potentially severe visceral involvement. Limited understanding of immunopathogenesis, in particular, the origin, phenotype, and function of T cells mediating disease across the spectrum of dtDHR severity, impedes clinical care.

Immune-mediated drug reactions are classified as types 1 through 4 according to the Gell and Coombs model of hypersensitivity (6). Type 4 reactions (delayed-type) typically begin days to weeks after exposure to inciting antigen, though reactions can occur more quickly if there is a history of prior antigen exposure. This timing is consistent with a T cell–mediated response. Indeed, T cells and molecules typically attributed to T effector cells are consistently detected in skin samples from dtDHR (4, 7–16). Moreover, there has been increasing recognition of HLA allele associations with specific drug:dtDHR (17, 18), further supporting T cells as protagonists. SJS/TEN research repeatedly has shown a cytotoxic CD8^+^ T cell predominance in skin and blister fluid (7–14). However, the CD8^+^ T cell subset or subsets mediating disease, their origin, and mechanism and location of activation remain unknown. Even the putative effector function by which CD8^+^ T cells mediate keratinocyte death is debated. Some studies support cytotoxic granule components (8, 10–12, 19), Fas:FasL (19, 20), or granulysin (13), and recently, CD8^+^ T cells have been proposed as triggering monocyte-mediated keratinocyte death (21). Data are more limited in DRESS and MDE, which is particularly noteworthy given MDE’s commonality. It is unclear whether they are CD4^+^ or CD8^+^ T cell mediated, Th1, Th2, or Th17 polarized, and/or cytotoxic (5, 22, 23).

Skin-resident memory T cells (TRMs) are a unique population of TRMs that reside long term in skin even during immunologic quiescence (24). Skin TRMs are increasingly implicated in the pathogenesis of inflammatory skin diseases, most notably allergic contact dermatitis (25), another form of delayed-type hypersensitivity reaction, and acute graft-versus-host disease (26), which clinically and histologically can present identically to dtDHR. Subsequently, a role for skin TRM in dtDHR has been surmised. Recent research supports that skin TRM are generated by dtDHR (27), but there are no studies to date investigating whether skin TRMs mediate disease. Knowing whether TRMs or other T cell subsets mediate disease is a critical step in illuminating the mechanism and location of pathogenic T cell activation and can lead to important clinical advancements, such as identifying biomarkers of disease progression, development of a test to identify culprit drugs, and discovery of an efficacious treatment, all of which are currently lacking.

DtDHR pathobiology is historically underresearched (1) due to 3 main barriers: (i) the rarity and acuity of severe disease impedes prospective sample collection, (ii) skin samples obtained for clinical purposes are typically formalin-fixed, paraffin-embedded (FFPE), which previously precluded extensive laboratory analysis, and (iii) there is a lack of adequate mouse models. Herein, we leveraged recent technical advancements to overcome these prior limitations to interrogate the origin, phenotype, and function of pathogenic T cells, in particular, skin TRM, in dtDHR.

## Results

### Retrospective analysis supports CD8^+^ T cell recruitment into SCAR but not MDE skin

**Figure 1:**
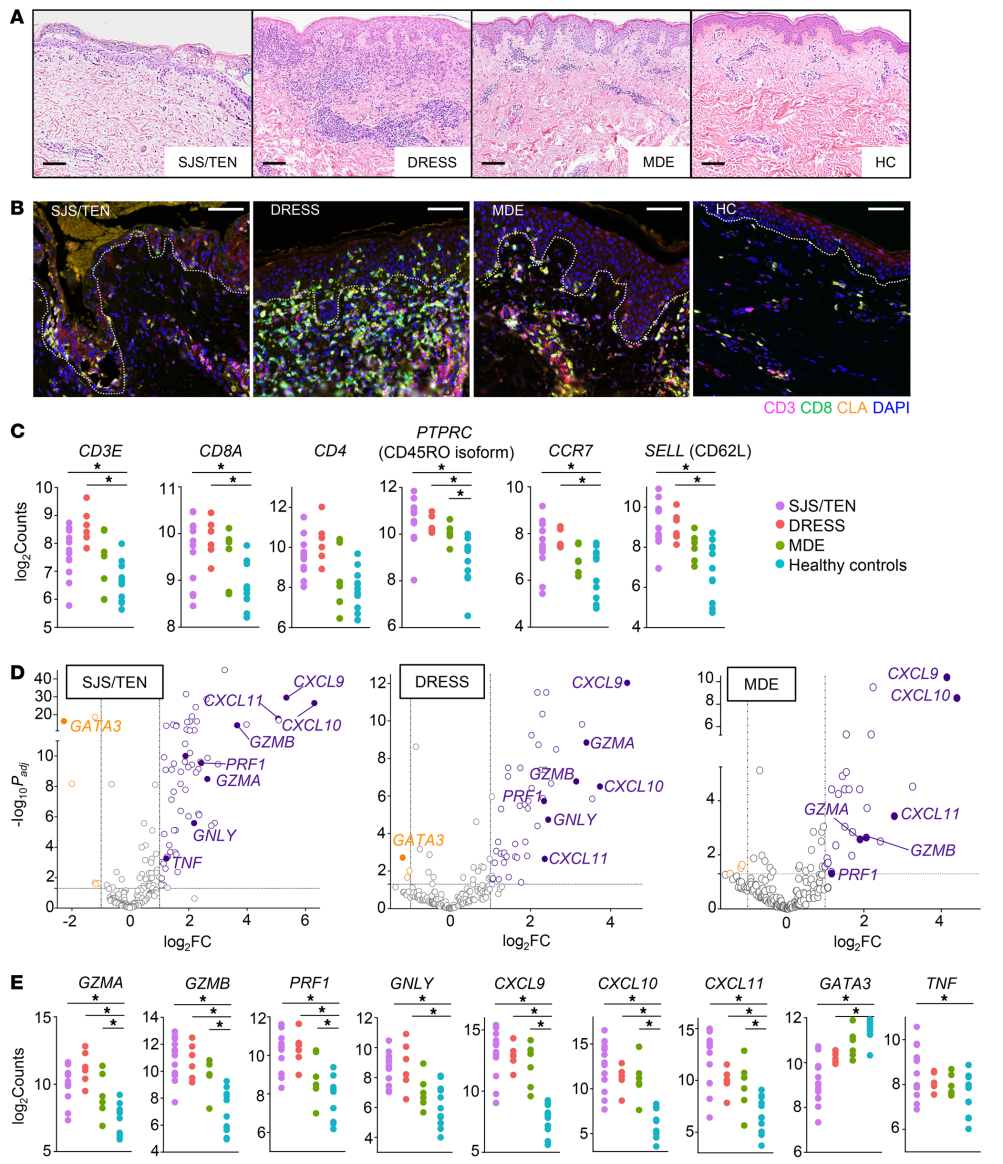
Retrospective skin sample analysis demonstrates variable T cell phenotypes and function across dtDHR severity. (**A**) Representative H&E images of dtDHR and healthy skin. Scale bars: 100 μm. (**B**) Immunofluorescent staining of dtDHR and healthy skin for CD3 (magenta), CD8 (green), and CLA (orange), with DAPI nuclear stain (blue). Scale bars: 100 μm. Gray dotted lines depict dermoepidermal junction. (**C**) The log_2_ counts of T cell phenotypic genes. (**D**) Volcano plots highlighting significantly differentially expressed functional markers in diseased versus healthy skin. (**E**) The log_2_ counts of functional markers. (**A** and **B**) *n* = 3–6 per group. (**C**–**E**) *n* = 13 SJS/TEN, 6 DRESS, 6 MDE, and 11 healthy controls. *Significance defined as absolute value │log_2_FC │≥ 1 and *P_adj_* < 0.05, DESeq2, Wald test.

We analyzed FFPE skin samples previously collected for clinical purposes from SJS/TEN, DRESS, and MDE patients and healthy controls. Histologic analysis demonstrated typical findings associated with dtDHR (Figure 1A) (4, 16). SJS/TEN was marked by full-thickness epidermal necrosis with pauci-inflammatory infiltrate and DRESS demonstrated a robust mononuclear infiltrate. The reaction pattern and infiltrate in MDE were variable, though the infiltrate was generally less dense. Immunofluorescence staining and microscopy confirmed the presence of skin-homing (CLA^+^), CD8^+^CD3^+^, and CD8^–^CD3^+^ T cell subsets (Figure 1B and Supplemental Figure 1A; supplemental material available online with this article; https://doi.org/10.1172/JCI178253DS1) in all forms of dtHDR. There was a marked predominance of CD8^+^ T cells within the epidermis and along the dermoepidermal junction in SJS/TEN, variable infiltrate of epidermal T cells in DRESS, and many fewer epidermal CD8^+^ or CD8^–^ T cells in MDE (Figure 1B and Supplemental Figure 1B).

Next, in situ bulk transcriptional profiling using a 200-gene panel (Supplemental Table 1) was performed on SJS/TEN, DRESS, MDE, and healthy FFPE skin samples fixed immediately upon biopsy. Given limited prior transcriptional analysis of all 3 forms of disease, primary analysis compared each form of dtDHR to healthy skin. Differential gene expression analysis (DGEA) demonstrated that both severe forms of disease had significantly upregulated transcription of *CD3E*, *CD8A*, *PTPRC* (isoform CD45RO), *SELL* (CD62L),and *CCR7*, suggesting potential proliferation and/or recruitment of memory CD8^+^ T cells from secondary lymphoid organs (SLOs) and/or blood (Figure 1C) (28). Comparatively, MDE lacked significant upregulation of these markers (Figure 1C), suggesting that T cells were neither heavily recruited into nor extensively proliferating within MDE skin, consistent with microscopic analysis. None of the 3 diseases demonstrated increased *CD69* or *ITGAE* (CD103), skin TRM markers (29–31) (Supplemental Table 1). However, TRM may have low proliferative potential (29, 32, 33), so increased gene expression would not necessarily be expected even if TRMs were activated.

DGEA suggested that all 3 forms of disease were Th1/Tc1 skewed. Genes for cytolytic granule components, *GZMA*, *GZMB*, and *PRF1*, and IFN-γ signature genes, *CXCL9*, *CXCL10*, and *CXCL11*, were significantly upregulated in all dtDHR (Figure 1, D and E). Analysis further demonstrated significantly increased transcription of *GNLY* in SJS/TEN and DRESS and of *TNF* in SJS/TEN (Figure 1, D and E), similarly to prior reports (13, 19).

Secondary DGEA was performed between dtDHRs. Supplemental Table 2 shows all differentially expressed genes between each comparison with adjusted *P* value (*P_adj_*) < 0.1 and │log_2_FC│ ≥ 1. A principal component analysis (PCA) lot demonstrated a clear separation of the 3 forms of dtDHR from healthy skin, yet considerable overlap among the 3 forms of dtDHR (Supplemental Figure 2A). SJS/TEN demonstrated a greater Th1/Tc1 skew compared with MDE, with increased fold change of *GNLY*, *GZMB*, *PRF1*, *IFNG*, and *CXCL11* and decreased *GATA3* (Supplemental Figure 2B). Transcription of cytotoxic molecules showed considerable overlap between SJS/TEN and DRESS, contrary to what might be assumed. Comparatively, *IL6*, which dampens Treg suppression of T effector cells (34, 35), was elevated in SJS/TEN, and *CCL18*, which promotes Treg recruitment (36), was lower in SJS/TEN (Supplemental Figure 2B). These data intimate that SJS/TEN might have reduced regulatory capacity compared with MDE and DRESS.

### Prospective analysis suggests differential cytotoxic CD8^+^ T cell versus Treg expansion and recruitment between SJS/TEN and MDE

To more deeply interrogate T cell subsets during active disease, we prospectively studied viable CD45^+^CD3^+^ T cells sorted from skin and blood using single-cell RNA-sequencing (scRNA-Seq) with cellular indexing of transcriptomes and epitopes by sequencing (CITE-Seq) (37) along with T cell receptor sequencing (TCR-Seq) of 3 SJS/TEN patients, 3 MDE patients, and 3 healthy controls (Supplemental Table 3). The prospective study focused on SJS/TEN and MDE, given the clearer divergence in immunologic milieu observed by histology and bulk transcriptional profiling between these 2 forms of dtDHR. Notably, MDE patient 2 had a robust skin reaction at risk for progression to severe disease with systemic involvement, so received high-dose systemic steroids with clinical improvement (did not progress), while MDE patients 1 and 3 had mild reactions.

**Figure 2:**
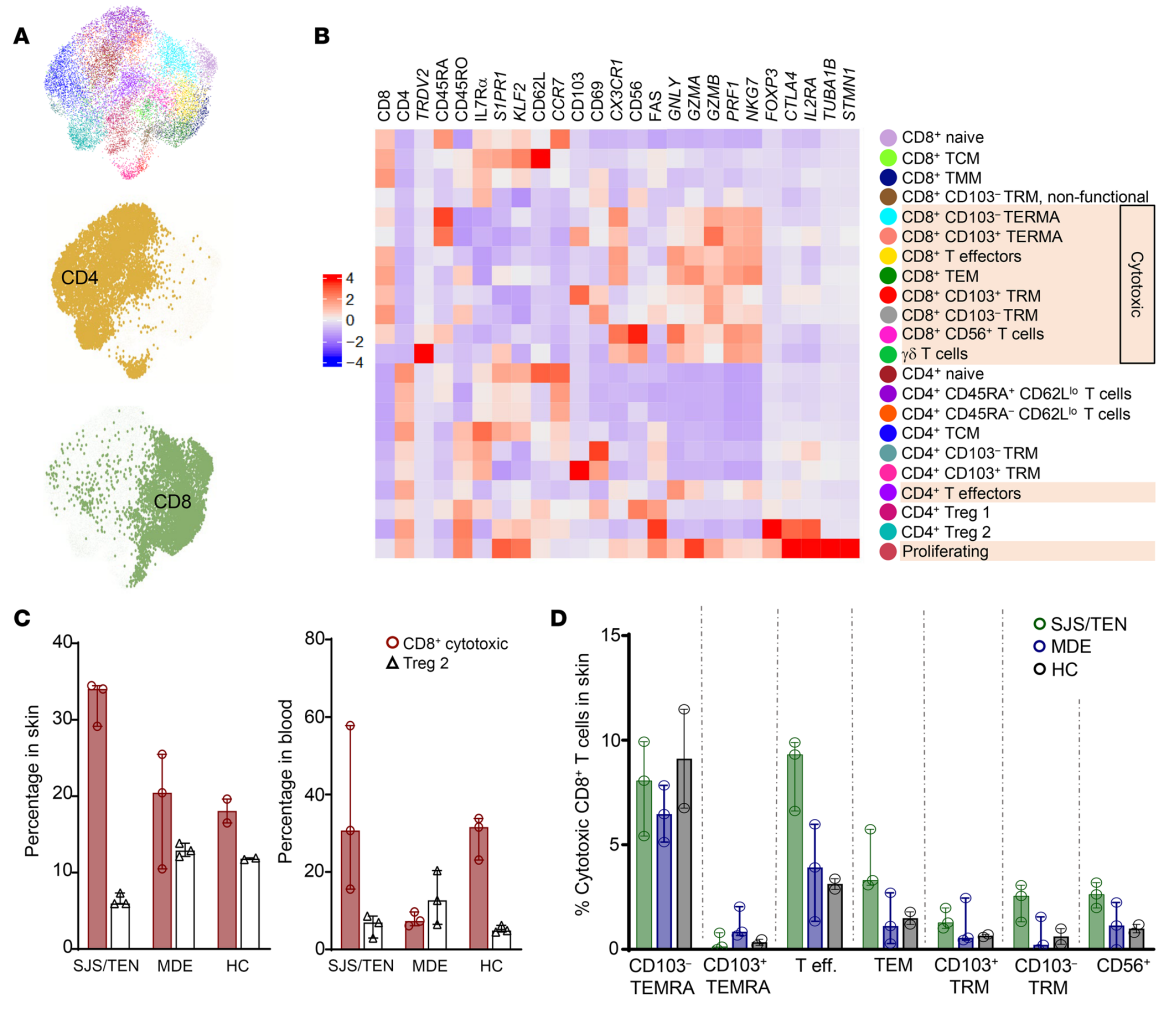
Prospective analysis by scRNA-Seq plus CITE-Seq reveals differential T cell populations across dtDHR. (**A**) UMAP of CD3^+^ T cells from 17 samples showing 22 clusters identified, with clear separation of CD4^+^ and CD8^+^ T cell subsets. (**B**) Heatmap identifying clusters by phenotypic and functional markers using both genes (italicized) and proteins (not italicized). Each box shows aggregate mean expression value of each marker of each cluster. (**C**) Median percentage plus range of cytotoxic CD8^+^ T cells and Treg2 in skin and blood of SJS/TEN, MDE, and healthy control (HC) patients. (**D**) Median percentage plus range of cytotoxic CD8^+^ T cells of total T cells in SJS/TEN, MDE, and healthy control skin across cytotoxic CD8^+^ T cell clusters identified from the heatmap.

Transcript and protein from skin and blood samples were integrated using the weighted nearest neighbors (WNN) method (38), resulting in 22 distinct cell clusters with clear separation between CD4^+^ and CD8^+^ T cells (Figure 2, A and B, and Supplemental Figure 3). The complete cluster marker list is shown in Supplemental Table 4. The addition of CITE-Seq markedly improved resolution of 7 key phenotypic markers (CD45RA, CD45RO, CD62L, IL-7Rα, CD69, CD103, and CD56) compared with scRNA-Seq alone (Supplemental Figure 4), consequently greatly improving cluster definition. Individual uniform manifold approximation and projection (UMAPs) of skin and blood using the WNN-based joint clustering method supported joint-clustering definitions (Supplemental Figure 5), and clustering of skin and blood performed separately further affirmed the WNN-based joint (skin plus blood) clustering (Supplemental Figures 6 and 7).

#### Cell-population analysis.

The total number and percentage of T cells in each cluster in each patient in skin and blood, fold change of percentage of T cells in each cluster in each patient’s skin and blood relative to healthy controls, and differential cell proportion analysis are shown in Supplemental Data Set 1. There was a trend toward higher percentages of several CD8^+^ T cell clusters in SJS/TEN versus MDE skin and blood, with multiple clusters significant by *P* value (Supplemental Data Set 1). The percentage of cytotoxic CD8^+^ T cells (identified using the mean normalized expression of *NKG7, GNLY, GZMA, GZMB*, and *PRF1*) was significantly elevated in SJS/TEN skin and blood compared with MDE (Figure 2C and Supplemental Data Set 1). Conversely, the percentage of Treg2 in skin was significantly lower in SJS/TEN than in MDE or healthy controls (Figure 2C and Supplemental Data Set 1), resulting in marked skewing of the cytotoxic CD8^+^ T cell/Treg ratio in SJS/TEN (Figure 2C). In skin, at the site of damage, cytotoxic CD8^+^ T cells in both SJS/TEN and MDE were distributed across multiple clusters, including CD8^+^ T effectors, CD8^+^ effector memory T cells (TEMs), CD8^+^CD56^+^ T cells, and terminally differentiated effector memory CD8^+^ T cells (TEMRAs), all classically considered recruited populations, as well as CD8^+^CD103^+^ and CD103^–^ skin TRM (Figure 2D), suggesting that multiple T cell subsets, both resident and recruited, could potentially be contributing to disease.

#### T cell subset functionality.

**Figure 3:**
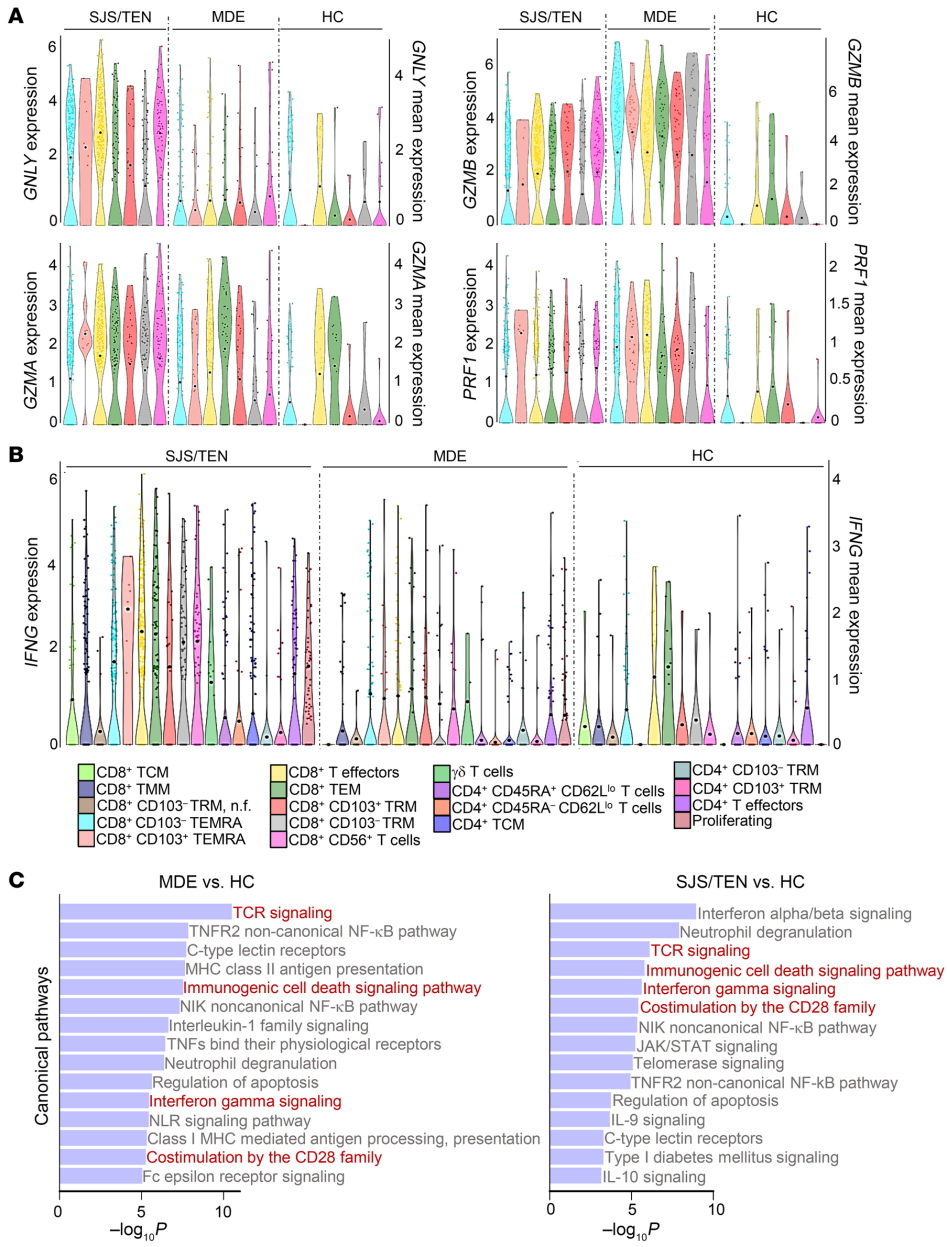
Multiple T cell subsets, including skin TRM, may be functional in SJS/TEN and MDE. (**A**) Violin plots showing cytotoxic markers *GNLY, GZMB, GZMA*, and *PRF1* in SJS/TEN, MDE, and healthy control skin across cytotoxic CD8^+^ T cell clusters. (**B**) Violin plot showing *IFNG* in SJS/TEN, MDE, and healthy control skin across all potential effector (nonnaive and non-Treg) clusters. (**A** and **B**) Violin plots show gene expression (left *y* axis) and mean expression (right *y* axis, and visualized by black dots). Cluster legend at figure bottom. n.f., nonfunctional. (**C**) Fifteen most significant canonical pathways by –log_10_*P* value with *z* score ≥ │2│, Fisher’s exact test, of skin CD8^+^ (CD103^+^ and CD103^–^) TRM clusters. Red text highlights pathways directly relevant to TRM activation and Th1/Tc1 function. There were no significant pathways between MDE and SJS/TEN CD8^+^ TRM clusters.

To investigate T cell subset functionality, pseudo-bulk DGEA (Supplemental Figure 8A), then cluster-specific single-cell DGEA (Supplemental Data Set 2, sheet 1), were performed on all genes. Pseudo-bulk DGEA confirmed a prominent CD8^+^ Th1/Tc1 signature in SJS/TEN. Th1/Tc1 gene transcription was thus examined at the single-cell level across individual T cell clusters visually (Figure 3, A and B) and with associated DGEA (Supplemental Data Set 2, sheet 2). *GNLY* was expressed significantly more at a single-cell level across multiple T cell clusters in SJS/TEN skin compared with MDE and healthy controls. Comparatively, cytotoxic granule components *GZMA*, *GZMB*, and *PRF1* were largely comparably transcribed between SJS/TEN and MDE skin. Both resident and recruited CD8^+^ T cell clusters transcribed cytotoxicity genes (Figure 3A), and along with recruited CD4^+^ T cell subsets, transcribed *IFNG* (Figure 3B). Gene expression varied across individual patients within groups (Supplemental Figure 9).

While CD8^+^ TRM clusters appeared to be activated in SJS/TEN and MDE skin (Figure 3, A and B), they made up a small proportion of cytotoxic CD8^+^ T cells compared with recruited populations (Figure 2D). Pathway analysis supported that CD8^+^ TRMs were activated in both SJS/TEN and MDE compared with healthy skin (Figure 3C), and on a single-cell level, CD8^+^ TRMs transcribed significantly more cytotoxicity genes in both SJS/TEN and MDE compared with healthy skin (Supplemental Data Set 2, sheet 3).

Conversely, pseudobulk DGEA demonstrated a Treg signature in MDE compared with SJS/TEN and healthy controls. It highlighted 2 potentially relevant genes, *CCR8*, a chemokine receptor that can bind to CCL18 (39), and *TNFRSF4* (OX40), which promotes Treg survival (40). This was not simply due to the increased percentage of Tregs in MDE, as on a single-cell level, Treg2 in MDE skin transcribed significantly more *CCR8* and *TNFRSF4* than in SJS/TEN skin (Supplemental Figure 8B and Supplemental Data Set 2, sheet 4).

#### Differential clonal expansion between SJS/TEN and MDE.

**Figure 4:**
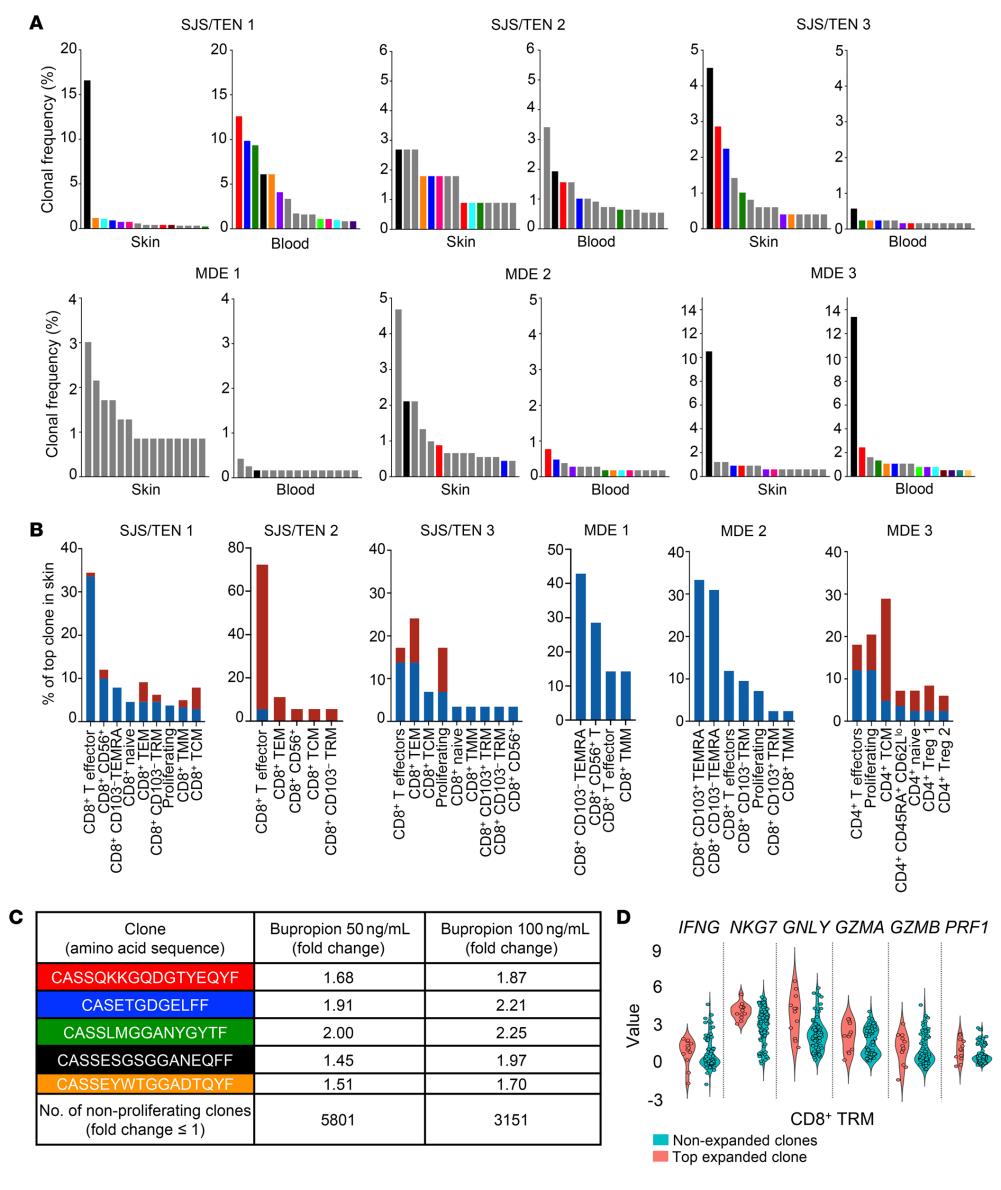
TCR-Seq identifies clonal expansion in blood of cytotoxic CD8^+^ T cells in SJS/TEN but not MDE. (**A**) Clonal frequency (percentage) of the top 15 clones in skin and blood of each dtDHR patient. Clones found in both skin and blood at any frequency of each patient are color coded (black is 1 clone, red is 1 clone, etc). Clones found only in skin or blood of an individual patient at any frequency are gray. (**B**) Bar graph showing percentage distribution across T cell clusters of the top clone in skin (blue). If that same clone was also found in blood, it is additionally shown in red. (**C**) Table showing fold change of clones in blood from SJS/TEN patient 1 cultured with suspected culprit drug, bupropion, at 2 concentrations compared with vehicle. The top 5 clones deemed expanded in blood in vivo (from **A**) are individually shown and color coded to match (in **C**). (**D**) Violin plot showing relative value expression of Th1/Tc1 markers in CD8^+^ TRM comparing the top expanded clone to all nonexpanded clones (defined as ≥3 consecutive clones of the same frequency) in SJS/TEN patient 1 skin.

The above cumulative findings raise the possibility of expansion and recruitment of cytotoxic CD8^+^ T cells into skin in SJS/TEN, but not MDE. To better assess T cell expansion in dtDHR, clonality analysis of every fully TCR-sequenced T cell in each sample was performed. The top 20 clones in skin and blood are detailed in Supplemental Data Set 3. Sequencing data from every sequenced clone of each patient’s skin and blood is publicly accessible (see *Data availability*). The productive frequency (percentage) of each of the top 15 clones in each sample was graphed, with clones detected in both skin and blood uniquely color coded, while clones limited to 1 tissue are shown in gray (Figure 4A). Since there is no clear definition of an actively expanding clone by clonal frequency assessed in an individual sample, we began with a stringent quantitative approach whereby clonal expansion was defined as 3× or more the frequency of nonexpanding clones (≥3 consecutive clones with the same frequency) within each skin sample. Using this initial approach, SJS/TEN patients 1 and 3 and MDE patients 1, 2, and 3 had expansion of 1 or more clones in skin, while healthy skin lacked expansion (Supplemental Figure 10A). We then compared the top clones between skin and blood in each patient. The top expanded clones in skin of SJS/TEN patients 1 and 3 and MDE patient 3 were each among the top clones in blood, suggesting that clonal expansion had occurred in SLOs/blood. The top clone in skin of SJS/TEN patient 2 (which did not meet the quantitative definition) was the second highest clone in that patient’s blood, raising the possibility of expansion in blood. Comparatively, the top expanded clones in skin of MDE patients 1 and 2 were not detected in blood, suggesting potentially skin-limited expansion (Figure 4A).

Given limitations of using clonal frequency alone to interpret active expansion, we capitalized on the scRNA-Seq plus CITE-Seq data obtained concurrently to ascertain the phenotypes of the top expanded cells, with the presumption that actively expanding cells should fall predominantly into activated clusters. In all 3 SJS/TEN patients, the top expanded clone in skin was a cytotoxic CD8^+^ T cell spanning highly functional clusters in both skin and blood (Figure 4B). The top expanded clones in MDE patients 1 and 2 skin were cytotoxic CD8^+^ T cells spanning highly functional clusters, but were not detected in blood. The top expanded clone in MDE patient 3 was in both skin and blood but was a CD4^+^ T cell that spanned Treg clusters (Figure 4B). Comparatively, the top clone in both healthy skin samples was a CD4^+^ T cell spanning nonfunctional clusters (Supplemental Figure 10B). Taken together, the data support that clonal expansion occurred at least at low levels in skin in all 6 patients, but clonal expansion of a cytotoxic CD8^+^ T cell clone occurred in SLOs/blood only in SJS/TEN.

Though the observed clonal expansion was likely drug specific, it is theoretically possible that the expanded T cells reacted to another antigen. Rarely, SJS/TEN can occur secondary to infection, most commonly *Mycoplasma* or herpes simplex virus (HSV) (2, 5). The clinical and histologic findings in MDE can occur secondary to virus alone or to the combination of drug plus virus (41). The 6 prospective study patients lacked signs/symptoms and/or tested negative for *Mycoplasma*, HSV, and other viral infection (Supplemental Table 3). Further, comparison of the patients’ TCR paired-chain sequences and HLA-A and -B alleles to those in the VDJdb (42) and McPAS-TCR (43) databases, to unpaired chain sequences if pairs were unavailable, or to all HLA-C, DR, DP, and DQ alleles reported in the databases revealed no overlapping sequences. These data argue against but do not rule out an infectious antigenic source.

Comparison of our patients’ TCR sequences to published sequences of expanded T cell clones in dtDHR (14, 44–47) revealed no shared sequences, though this was unsurprising, as there was no overlap between the presumed culprit drug and HLA-A or HLA-B allele of our study patients with the published data. Confirmation of drug specificity is challenging to demonstrate in the lab, as drug reactivity assays have historically poor sensitivity (48). Based on our data, we posited that clonal expansion could serve as a readout for drug reactivity. Using residual PBMCs from SJS/TEN patient 1, we performed a mixed lymphocyte reaction in the presence or absence of the presumed culprit drug and used high-throughput TCR-β sequencing as a readout. The top expanded clones in blood by TCR-Seq (Figure 4A) expanded ex vivo in the presence of drug 1.7-fold or more compared with vehicle control, while thousands of clones did not expand in the presence of drug (fold change ≤ 1), supporting that expanded clones were drug reactive (Figure 4C).

Finally, to further understand the apparent incongruity in TRM percentage and functionality, we compared transcription of pertinent Th1/Tc1 molecules between the most expanded clone and all nonexpanded clones in skin CD8^+^ TRM clusters in SJS/TEN patient 1. Results demonstrated that a population of nonexpanded clones with skin TRM phenotype transcribed functional molecules (Figure 4D), supporting that skin TRM can be functional despite limited proliferative capacity and thus limited abundance (29, 32, 33).

### Skin TRM may mediate MDE in the absence of circulating T cells

**Figure 5:**
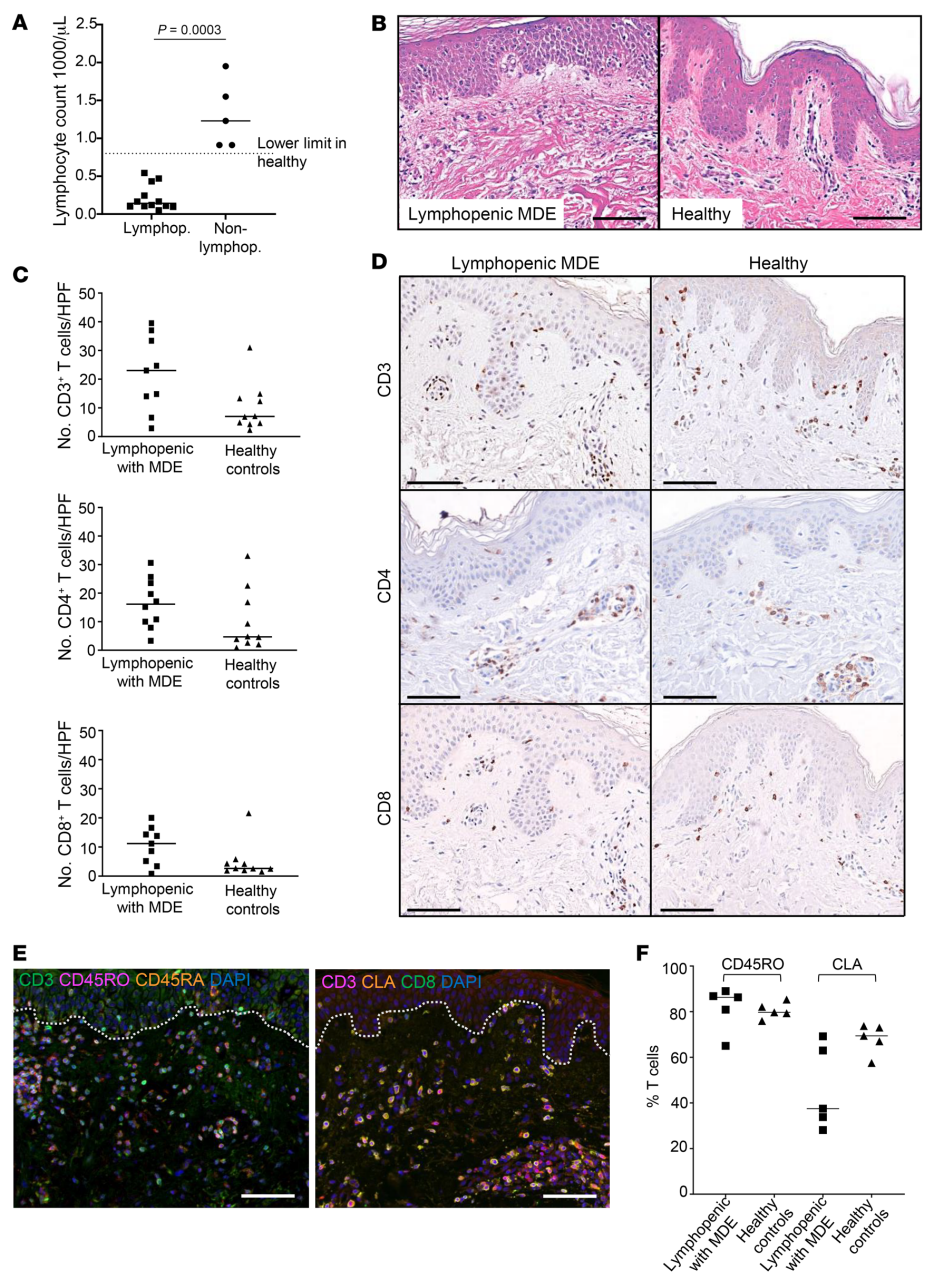
Human skin TRM may be sufficient to mediate MDE. (**A**) Number of lymphocytes in peripheral blood of MDE patients with or without lymphopenia. Lower limit of healthy depicted as dotted line. (**B**) Representative H&E images from a lymphopenic MDE patient and healthy control demonstrating similar mononuclear infiltrate in skin. (**C**) CD3^+^, CD4^+^, and CD8^+^ T cell count per high-powered field (HPF) by immunohistochemistry in lymphopenic MDE patient versus healthy skin. (**D**) Representative immunohistochemistry images of CD3^+^, CD4^+^, and CD8^+^ T cells from lymphopenic MDE patient and healthy skin. (**E**) Representative immunofluorescence staining in skin of lymphopenic MDE patient for CD3 (magenta), CD45RO (green), and CD45RA (orange) (left image) and CD3 (magenta), CLA (orange), and CD8 (green) (right image), with DAPI nuclear stain (blue). (**F**) Percentage of CD45RO^+^CD3^+^ T cells and CLA^+^CD3^+^ T cells per HPF of lymphopenic MDE patient compared with healthy skin. Scale bars: 100 μm (**B**, **D**, and **E**). (**A**, **C**, and **F**) Lines show median. Significance defined as *P* < 0.05, 2-tailed Mann-Whitney *U* test. Only *P* < 0.05 shown.

The above cumulative data implicate but do not clearly show a functional role for skin TRM in disease pathogenesis. Further, some degree of T cell recruitment from circulation into skin was evident in at least some MDE cases, though even in TRM-mediated diseases, non-TRM subsets can be recruited into and/or circulate through skin due to the inflammatory milieu (49–52). We aimed to directly test the functional contribution of TRM to MDE, with the ideal model system containing only TRM. Anecdotally, MDE can develop in patients that are severely lymphopenic, insinuating that skin TRM alone can mediate MDE. We retrospectively identified 12 patients with clinically diagnosed MDE despite lymphopenia secondary to varying chemotherapy regimens for acute myelogenous leukemia (Supplemental Table 5). Importantly, we specifically chose patients with lymphopenia that was secondary to chemotherapy, since patients with dtDHR may develop lymphopenia as a result of drug reactions (53, 54). Also notably, human skin TRMs have been shown to survive varying chemotherapy regimens (26, 55, 56) and, though lymphopenic patients may have aberrant immune systems from their underlying disease, they often react to the same drugs and have MDE clinically and histopathologically indistinguishable from that of nonlymphopenic patients (morbilliform eruption and spongiotic and/or interface pattern, respectively). Despite markedly reduced circulating lymphocytes (Figure 5A), histologic assessment identified lymphopenic MDE patients with minimal mononuclear cell infiltrate, largely comparable to healthy skin (Figure 5B and Supplemental Figure 11), where the majority of T cells are TRMs (29). Quantification by immunohistochemistry demonstrated similar numbers of CD3^+^, CD4^+^, and CD8^+^ T cells between lymphopenic MDE patients and healthy controls (Figure 5, C and D). A subset of lymphopenic MDE patients had numbers of T cells (Figure 5, C and D) with a CD45RO^+^ skin-homing (CLA^+^) phenotype (Figure 5, E and F, and Supplemental Figure 12) comparable to healthy skin, arguing against recruitment of T cells from circulation into skin in at least a subset of patients. These data support that skin TRM may be sufficient to mediate MDE despite the absence of circulating T cells.

### Skin TRM can mediate MDE-like disease in mice

**Figure 6:**
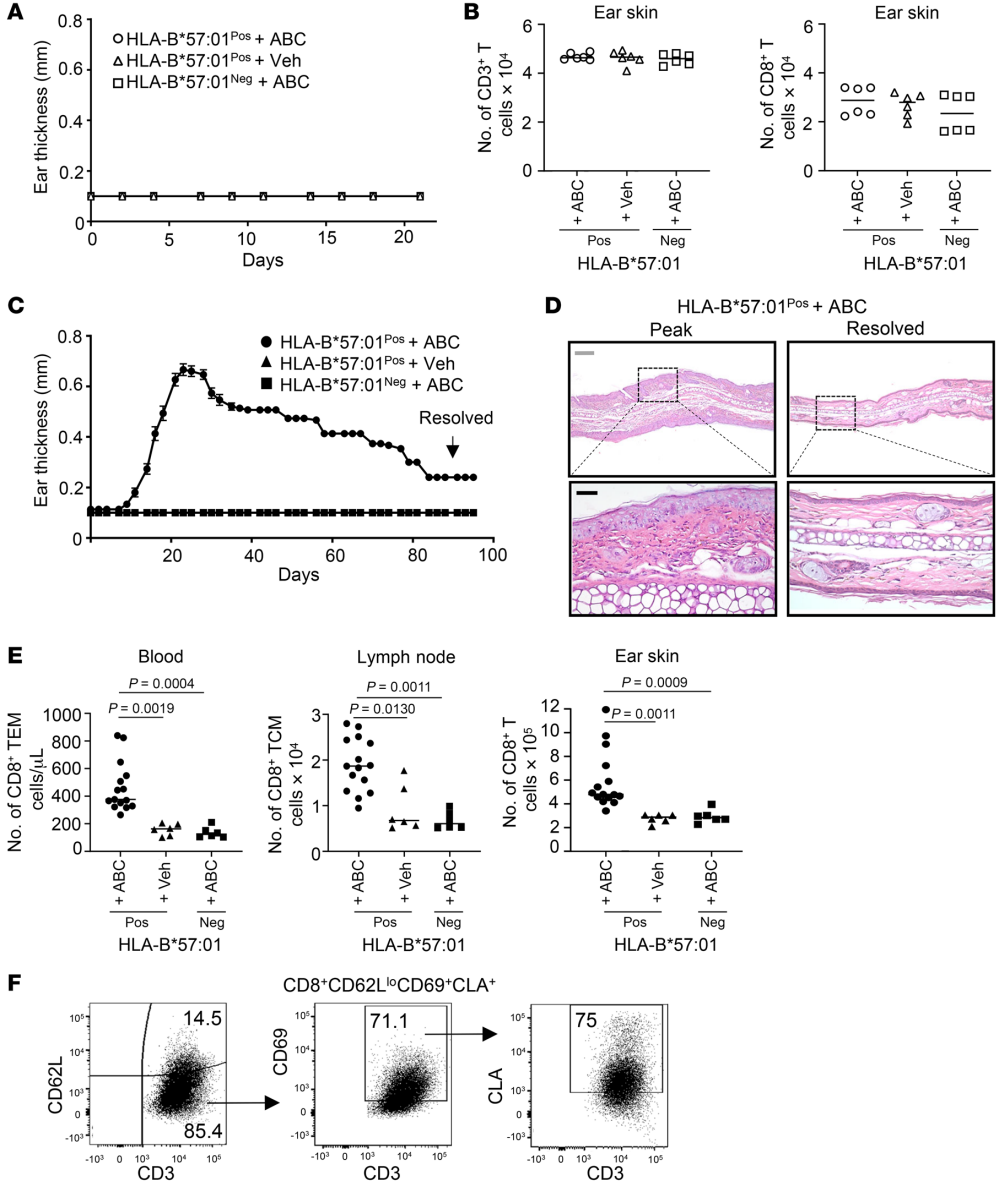
Drug-specific skin TRMs are generated in mouse skin after disease resolution. HLA-B*57:01^pos^ and HLA-B*57:01^neg^ mice treated with systemic drug alone did not develop skin inflammation by (**A**) ear thickness (mean with SEM shown) or (**B**) total number of CD3^+^ T cells and CD8^+^ T cells in ear skin by flow cytometry. Mice treated with systemic and topical drug developed skin inflammation that slowly resolved by 90 days after treatment as measured by (**C**) ear thickness (mean + SEM shown) and (**D**) histology. Scale bars: 200 μm (gray); 50 μm (black). (**E**) Total number of CD8^+^ TEMs (CD44^hi^CD62L^lo^) in blood, TCMs (CD44^hi^CD62L^hi^) in LN, and total CD8^+^ T cells in ear skin quantified by flow cytometry (gated on CD3^+^CD8^+^ T cells). (**F**) CD8^+^ T cells in resolved ear skin show a TRM (CD62L^lo^CD69^+^CLA^+^) phenotype by flow cytometry. Plots gated on CD3^+^CD8^+^ T cells. (**A**–**F**) Each experiment repeated at least twice. Pooled results from 2 independent experiments shown. (**B** and **D**) Lines show median. Significance defined as *P* < 0.05, Kruskal-Wallis test followed by Dunn’s multiple-comparisons test between experimental and each control group.

Given potential limitations of the lymphopenic patient study, we aimed to test the role of skin TRM versus recruited populations in MDE-like disease in a mouse model in which all other variables are controlled (i.e., all mice have a healthy immune system). HLA-B*57:01 predisposes patients taking the drug abacavir (ABC) to dtDHR (57, 58). Cardone et al. previously generated HLA-B*57:01Tg mice that developed CD8^+^ T cell–mediated ear dermatitis in response to topical plus systemic ABC exposure coupled with CD4^+^ T cell depletion (59). In our hands, despite depletion of CD4^+^ T cells, treatment with systemic ABC alone failed to induce a reaction (Figure 6, A and B). Based on our human data, we hypothesized that this was because TRMs, in particular skin TRM, are necessary to mediate disease and naive mice lack a legitimate TRM pool, including skin TRM (60). We modified the original mouse model to test this hypothesis. We treated HLA-B*57:01^pos^ mice or HLA-B*57:01^neg^ littermate controls depleted of CD4^+^ T cells with ABC or vehicle systemically and topically to ear skin. Experimental mice developed a cytotoxic CD8^+^ T cell–mediated skin-limited reaction that was HLA-B*57:01 and drug dependent (Supplemental Figure 13). In this setting, CD8^+^ T cells were primed in SLOs and migrated through blood into skin to mediate disease (Supplemental Figure 13). Drug-induced dermatitis slowly resolved by day 90 (Figure 6C). Ear thickness decreased, but did not return to baseline, as ears were scarred; however, active inflammation resolved based on clinical and histologic evaluation (Figure 6D). Despite the absence of active inflammation, ear skin of HLA-B*57:01^pos^, drug-treated mice demonstrated a CD8^+^ T cell population expressing CD62L^lo^CD69^+^CLA^+^ consistent with skin TRM (Figure 6, E and F). Concurrently, TEMs (CD62L^lo^CD44^hi^) were observed in blood and TCMs (CD62L^hi^CD44^hi^) in LNs (Figure 6E).

**Figure 7:**
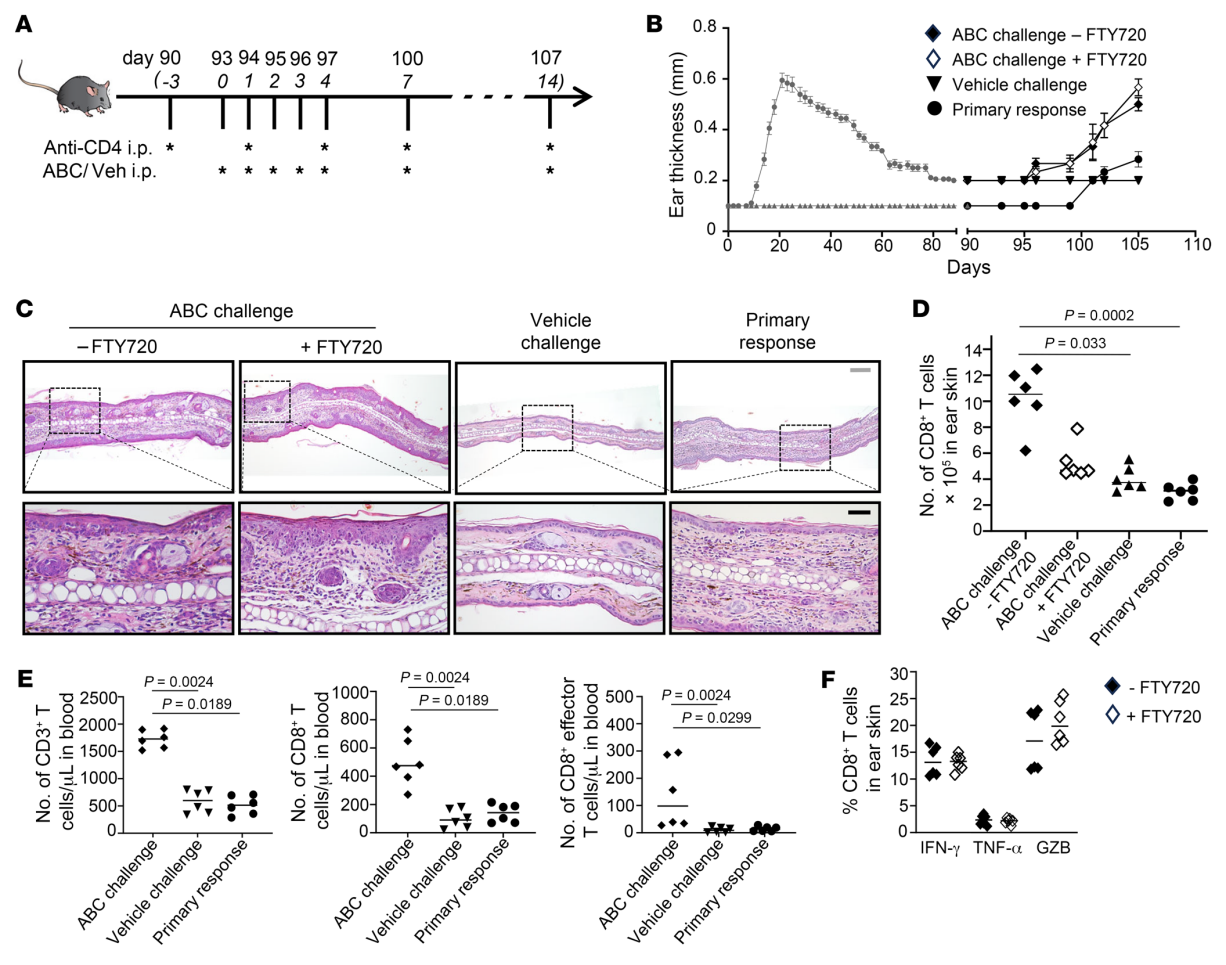
Skin TRMs mediate an MDE-like reaction in mice in the absence of circulating T cells. (**A**) Schematic of drug challenge experiment. Endpoint: 107 days. (**B**) Ear thickness (mean + SEM shown). (**C**) Representative histology. Scale bars: 200 μm (gray); 50 μm (black). (**D**) Total number of CD8^+^ T cells in ear skin. (**E**) Number of CD3^+^ T cells, CD8^+^ T cells, and effector CD8^+^ T cells (CD44^hi^CD62L^lo^) in blood. (**F**) Percentage of functional CD8^+^ T cells in ear skin of mice treated or not with FTY720. (**A**–**F**) Each experiment was repeated at least twice. Pooled results from 2 independent experiments shown. (**D**–**F**) By flow cytometry. Lines show median. (**D** and **E**) Significance defined as *P* < 0.05, Kruskal-Wallis test followed by Dunn’s multiple-comparisons test. (**F**) Nonsignificant, *P* > 0.05; 2-tailed Mann-Whitney *U* test.

To confirm that drug-reactive TRMs were generated by this method and investigate whether these TRMs could mediate a true drug allergy, mice underwent in vivo drug challenge. At day 90, HLA-B*57:01^pos^ mice previously treated with ABC or vehicle were now treated systemically with ABC or vehicle without topical treatment (Figure 7A). Mice containing drug-reactive TRMs developed an MDE-like reaction upon drug challenge, marked by increased ear thickness and clinically and histologically evident dermatitis, faster than the primary drug-exposed mice, consistent with a TRM response (Figure 7, B and C). This reaction was drug specific, as HLA-B*57:01^pos^ mice previously immunized against drug but now challenged with vehicle failed to develop a reaction. The reaction included expansion of CD8^+^ T cells in LN and blood, with migration into skin, indicating that this reaction was not purely a local immune response or percutaneous reaction (Figure 7, D and E).

A subset of drug-challenged mice was concurrently treated with FTY720, an S1PR1 agonist that prevents egress of T cells from lymphoid organs (61, 62). These mice had markedly reduced numbers of circulating CD8^+^ T cells (Supplemental Figure 14), yet developed dermatitis only slightly delayed compared with non-FTY720–treated drug-challenged mice, who had the ability to recruit T cells to skin from SLOs (Figure 7, B and C). Moreover, FTY720-treated mice had a slightly reduced number of CD8^+^ T cells yet similar percentages of functional CD8^+^ T cells in ear skin compared with non-FTY720–treated mice (Figure 7, D and F), further supporting that the main mediators of disease were skin TRMs. These data directly parallel the observations in lymphopenic patients showing that skin TRMs may be sufficient to mediate skin-limited reactions.

## Discussion

The contribution of skin TRMs versus other T cell subsets to dtDHR has been speculated on but not tested. A multimodal translational approach provided tremendous insight into this question. Most strikingly, SJS/TEN was seemingly marked by cytotoxic CD8^+^ T cells spanning multiple T cell subsets, resident and recruited, along with clonal expansion in skin and blood. Comparatively, MDE had clonal expansion in skin of cytotoxic CD8^+^ T cells with overall low-level recruitment from blood. In support, Villani et al. observed clonal expansion in blister fluid/skin and blood in the majority of TEN patients, but not MDE patients, assayed by high-throughput TCR-Seq (14), and Pan et al. detected clonal expansion of drug-reactive cytotoxic CD8^+^ T cells in blister fluid and blood in carbamazepine-induced SJS/TEN by next-generation sequencing (45). Though DRESS cases were not included in our prospective study, Picard et al. previously observed expansion of CD8^+^ T cells in blood of DRESS patients, affirming that CD8^+^ T cell expansion in blood occurs in both forms of SCAR (15). Comparatively, our data implicated skin TRMs as potential protagonists in MDE, and our observations in humans and mice unable to effectively recruit T cells into skin provided functional evidence that skin TRMs may be sufficient to mediate MDE. These findings make teleological sense, since SCAR patients suffer from systemic involvement while MDE is skin limited.

Aberrant Treg development, recruitment, and/or function has been implicated in SJS/TEN pathogenesis (63–66), while DRESS reportedly shows increased Tregs (67). Our findings highlighted a reduced percentage of CD4^+^ Tregs in SJS/TEN versus MDE and healthy skin, and transcriptional data pointed toward potentially impaired Treg recruitment, development, and/or survival in SJS/TEN compared with DRESS and MDE. Our results require confirmation at the protein level with functional experimentation, but are important to pursue, as a defective Treg response may be critical for the blistering observed in SJS/TEN but not DRESS.

The variability in T cell responses among MDE patients warrants further investigation in a larger prospective study. MDE patient 2 demonstrated a clonally driven CD8^+^ cytotoxic T cell response in skin but not blood and did not progress clinically to SCAR, insinuating that dtDHR may begin in skin, with systemic drug-reactive CD8^+^ T cell activation a key step in progression. A prospective study sequentially sampling MDE patients that do or do not progress is necessary to address this, but is critically important as it implies that (i) cytotoxic CD8^+^ T cell expansion in blood could serve as a biomarker of dtDHR progression and (ii) early intervention with high-dose systemic steroids may be able to halt progression. In support of the former, Villani et al. showed that clonal expansion in blood of TEN patients at disease onset correlated with final disease severity (14). Argument for the latter is strengthened by evidence that high-dose systemic steroids can abort recurrence of SCAR upon culprit drug rechallenge (68).

scRNA-Seq plus CITE-Seq plus TCR-Seq proved a powerful platform, as it revealed an incredible breadth of T cell phenotypic subsets in diseased skin. The addition of CITE-Seq markedly facilitated phenotypic identification of T cell subsets by allowing discrimination between the isoforms CD45RA and CD45RO and improving resolution of CD62L, IL7Rα, CD69, CD103, and CD56. A challenge in interpreting the scRNA-Seq plus CITE-Seq results was defining clusters, including skin-resident versus recruited populations. Though we relied on classical definitions of T cell subsets (28, 29, 32, 69, 70), current definitions are likely imperfect, (71) particularly during active inflammation, as T cell subsets may be more plastic than commonly credited (71). For example, we observed a small population of skin TRMs in circulation, corroborating recent reports that TRMs can exit peripheral tissues and enter circulation (72–74). Additionally, though TEMRAs are typically considered to be a circulating T cell subset, we identified a small population of cytotoxic TEMRAs expressing CD103 in skin and blood. CD103 mediates lymphocyte retention in epithelial tissues through binding to E-cadherin (75). Cluster analysis placed CD8^+^CD103^+^ TEMRAs adjacent to skin TRMs, suggesting this TEMRA subset was possibly skin resident. Finally, the top expanded clone in MDE patient 2 skin spanned clusters classically defined as recruited, yet that clone was not identified in blood, raising the possibility that the observed phenotypes were generated from an activated TRM. To this point, though expanded clones likely arose in SLOs and migrated through blood into skin, we cannot rule out that clones expanded in skin, then migrated into blood. We also cannot exclude the possibility that CD8^+^ T cell expansion and contraction already occurred in MDE at the time of sampling, though this is seemingly less likely given the cumulative data and that samples were collected during active disease. The scRNA-Seq plus CITE-Seq study was further limited by small sample size, and functional assessment was largely constrained to transcriptional data. Larger prospective studies, ideally with sequential sampling starting early in disease and using high-parameter flow cytometry with effector molecule staining, are likely necessary to overcome these limitations.

The lymphopenic patient study also had limitations. Microscopic analysis was performed on a small sample size. The patients had aberrant immune systems due to their underlying disease, though importantly, dtDHR in lymphopenic patients mirrors that of nonlymphopenic patients, and modeling using mice with intact immune systems comparable across experimental and control groups affirmed the human data. While the findings support that skin TRMs are sufficient to mediate MDE, it is possible that some degree of recruitment occurred even in patients with profoundly reduced circulating T cells or potentially that non-T cells contributed to or were even causal in disease.

Regardless of these limitations, this study’s findings illuminate several noteworthy aspects of immunopathogenesis with potentially important implications for both the clinic and fundamental immunology. Moreover, this study demonstrates that a multimodal, innovative approach spanning bedside to bench can overcome research obstacles in difficult-to-study diseases.

## Methods

### Sex as a biological variable.

Both sexes were included in human and mouse studies. Similar findings are reported for both sexes.

### Human studies.

Retrospective analysis was conducted on FFPE skin samples of SJS/TEN, DRESS, and MDE from adult and pediatric patients from January 1, 2000, to December 31, 2016, from BWH, BCH, and Massachusetts General Hospital. All cases were clinically diagnosed as dtDHR by board-certified dermatologists, had pathology consistent with the diagnosis read by board-certified dermatopathologists, and were vetted by a second board-certified dermatologist with expertise in dtDHRs. Alternative pathology or clinical diagnoses, cases lacking sufficient clinical data to confirm diagnosis, and cases of SJS (<10% body surface area blistered) were excluded to eliminate potential for misdiagnosis. A second patient cohort from BWH was obtained by searching pathology cases of MDE in patients that were lymphopenic (<1,000 lymphocytes/μL) at the time of skin biopsy.

Patients with clinically confirmed SJS/TEN or MDE were prospectively enrolled at BWH. A 6 mm punch biopsy, 40 mL peripheral blood, and medical record data were collected on each patient. Human skin discarded during plastic or dermatologic surgeries and PBMCs from blood bank leukopacks served as healthy controls.

### Skin staining and microscopy.

FFPE skin sections 5 to 6 μm thick were baked, deparaffinized, and rehydrated. H&E staining was carried out by standard technique. For immunohistochemistry/immunofluorescence staining, sections underwent antigen retrieval at 96°C, blocking of nonspecific protein binding, and staining. Primary antibodies used were as follows: CD103 (PA0374, Leica; EP206), CD3 (A0452, Dako; polyclonal), CD45RO biotinylated (304202, BioLegend; UCHL1), CD45RA (158-4D3, Novus; NBP2-15193), CD4 (104R-24, Cell Marq; EP204), CD8 (M7103, Dako; C8/144B), and CLA (321302, BioLegend; HECA-452). Secondary antibodies used were as follows: (BioLegend) AF555 goat anti-mouse IgG (Poly4053; 405324), AF555 donkey anti-rabbit IgG (Poly4064; 406412), AF488 streptavidin (405235); (Invitrogen) AF647 goat anti-mouse IgG (polyclonal; A-21236), AF647 goat anti-rabbit IgG (polyclonal; A21245) and AF488 goat anti-rat IgM (polyclonal; A21212); (Vector Laboratories) anti-mouse IgG (MP-7602) or anti-rabbit IgG (MP-7601) peroxidase ImmPRESS excel amplified polymer staining kit (with ImmPACT DAB) (Vector Laboratories). Sections were counterstained with DAPI or hematoxylin, then imaged using the Mantra Quantitative Pathology Workstation and analyzed using InFORM analysis software, version 2.3 (Akoya Biosciences).

### Bulk transcriptional profiling.

RNA was extracted from FFPE skin scrolls using the RNeasy Micro Kit (QIAGEN). Total RNA quantity and quality were measured using the BioDrop DUO spectrophotometer (Fisher Scientific), and a subset of samples was further evaluated using fragment analysis (Agilent Bioanalyzer, RNA NanoChip). RNA was concentrated as needed (RNA Clean & Concentrator Kit, Zymo Research). Samples were analyzed using a 200 gene custom designed panel (Supplemental Table 1) from NanoString Technologies on an nCounter Digital Analyzer.

Data quality was assessed using NanoStringQCPro, version 1.18.0 (76), and custom R code. Proportion of fields of view successfully counted, binding density, noise threshold, expression of positive and negative control genes, and expression of endogenous and housekeeping genes were evaluated. Samples found to be suboptimal were removed from analysis. Raw counts were normalized with the geometric mean using positive controls, then using a subset of housekeeping genes selected with an expression above a limit of detection (defined as mean expression of the negative control genes plus 2 times the SD) and a mean value higher than 200 (value selected empirically based on average housekeeping expression). Any gene expressed below this limit was removed. Differential gene expression was performed with DESeq2, version 1.26.0, R package (77). *P_adj_* values were estimated using the FDR to correct for multiple comparisons. Primary analysis compared each dtDHR to healthy controls. │log2FC│ ≥ 1, *P_adj_* < 0.05 was considered significant. Secondary analysis compared among the 3 forms of dtDHR. │log2FC│ ≥ 1, *P_adj_* < 0.1 was considered significant. All analyses were performed using R (version 3.6.0). Gene expression and volcano plots were made using GraphPad Prism, version 9.

### Prospective sample processing and staining.

Upon collection, skin biopsies were halved and each half frozen in 500 μL Cryostor CS10 cryopreservation media (07930; StemCell Technologies) in liquid nitrogen (LN2). PBMCs were collected from blood by Ficoll gradient and frozen in FBS plus 10% DMSO in LN2. Skin and PBMC samples from 3 SJS/TEN patients, 3 MDE patients, and 6 healthy control patients (3 healthy control skin and 3 healthy control blood that were not paired) were processed for scRNA-Seq plus CITE-Seq (antibody-derived tags [ADT]) plus TCR-Seq. Both frozen skin biopsy halves were thawed for 30 seconds in a 37°C water bath, rinsed in PBS, then thawed in 100% FBS on ice for 30 minutes. Skin samples were then rinsed in PBS, cut into small pieces, and incubated with Human Whole Skin Dissociation Kit Without Enzyme P (130-101-540; Miltenyi Biotec) for 2 hours 20 minutes at 37°C with agitation. Samples were washed in RPMI 1640 plus 10% FBS and centrifuged 400*g*, 4°C, 5 minutes). Skin was then disaggregated over a 70 μm filter, rinsed with RPMI 1640 plus 10% FBS, and centrifuged (400*g*, 4°C, 5 minutes). PBMCs were thawed at 37°C and washed with RPMI 1640 plus 10% FBS, then PBS. Cell pellets were resuspended in cold PBS. Cell counts and viability were determined by trypan blue.

Samples were stained with Zombie NIR Viability Dye according to the manufacturer’s instructions (423106; BioLegend), then washed with hash cell staining buffer (420201; BioLegend). Nonspecific antibody binding was blocked by 5% Fc Receptor Blocking Solution (422301; BioLegend) for 10 minutes on ice. Cells were stained with anti-human CD3, anti-human TotalSeq-C hashing antibodies (BioLegend) as previously described (78), and TotalSeq-C antibodies (BioLegend) (anti-human CD4, CD8, CD45RA, CD45RO, CD62L, CCR7, CD127, CD103, CD69, CD107a, FAS, CD56 and CD335) for 30 minutes on ice. Cells were then washed with cell-staining buffer and resuspended in sorting buffer (1× PBS, 2.5 mM EDTA, 25 mM HEPES, 1% FBS) for sorting using a FACSAria Fusion Cell Sorter (BD Biosciences). Live CD45^+^CD3^+^ T cells were collected into PBS plus 0.4% BSA for sequencing.

### Single-cell 5′ mRNA-Seq and analysis.

Single-cell RNA-Seq experiments were performed by the BWH Center for Cellular Profiling Core. Sorted viable CD45^+^CD3^+^ T cells from 3 skin and 3 blood samples were pooled and resuspended in 0.4% BSA in PBS at a concentration of 1,000 cells/μL, then loaded onto a single lane (Chromium chip A, 10x Genomics) followed by encapsulation in a lipid droplet (Single Cell 5′Kit V1, 10x Genomics), then by cDNA and library generation according to the manufacturer’s protocol. Three runs were performed with a total of 18 specimens.

A 5′ mRNA library was sequenced to an average of 50,000 reads per cell, and V(D)J library and hashtag oligo (HTO) library sequenced to an average of 5,000 reads per cell, using Illumina Novaseq. 10x Genomics reads were processed with Cell Ranger, version 3.1, for gene expression (using GRCh38 as reference genome), CITE-Seq, and hashtag oligo counts. Demultiplexing of pooled samples was performed using the Seurat R package (version 4.0, Satija Lab) (79) along with R, version 4.0.5. Raw HTO counts were normalized with centered log ratio (CLR) transformation; then HTODemux function was used to demultiplex pooled samples and to filter out (i) negative cells with low HTO unique molecular identifiers (UMIs) and (ii) multiplet cells with high HTO UMIs (78). Each of the 3 pooled samples was repeatedly demultiplexed, and 18 samples were separated into distinct Seurat objects. We preprocessed TCR-Seq reads with Cell Ranger, version 6.0.1, to call clonotypes. We used the djvdj package (https://github.com/rnabioco/djvdj) to import processed TCR-Seq reads into R.

Downstream analysis of the preprocessed scRNA data from 18 samples was performed using the Seurat R package, version 4 (38). To elucidate the T cell heterogeneity within the blood and skin samples, whose transcripts and surface protein were jointly assayed, we implemented a combination of standard integration and WNN analysis workflows of Seurat package (38).

Cells with (i) less than 1,000 or more than 20,000 total RNA UMI count, (ii) with more than 10,000 ADT UMI counts, or (iii) whose total mitochondrial (MT) gene expression counts exceeded 20% of their total UMI counts were filtered out. RNA and ADT assays from all 18 samples were log normalized and CLR normalized, respectively. CD4^+^CD8^+^ doublets and double positives were filtered out by removing cells having both more than 0.75 and 1 CLR normalized CD4 and CD8 ADT UMI counts, respectively. One healthy skin sample was removed from analysis given Seurat integration workflow failing due to low number of cells. This filtering step resulted in a scRNA data set of 15,084 cells.

The 2,000 most highly expressed genes in each of the 17 samples were detected using the variance stabilizing transformation (vst) method after ribosomal gene signatures were excluded from downstream analysis. We applied canonical correlation analysis on both RNA and ADT assays using 12 and 30 dimensions, respectively. This step of batch correction across 17 samples resulted in 2 separate assays for RNA and ADT. We scaled and then dimensionally reduced both integrated RNA and ADT assays before constructing the multimodal (combined RNA and ADT) shared nearest neighbor graph with the FindMultiModalNeighbors function (38). We used the resulting WNN graph to cluster the integrated data set and to build a 2D UMAP reduction for visual analysis.

To detect the cellular heterogeneity of the integrated blood and skin data sets, we used the FindCluster function with resolution parameters ranging from 0.4 to 1.8, using the graph-based smart local moving algorithm (80). We conducted marker analysis using the FindAllMarkers function (with a log_2_FC threshold of 0.25 and for genes that are detected in more than 10% of all cells) to test for significantly highly expressed genes and surface proteins in each cluster against all other clusters with Wilcoxon’s rank-sum test. Separately conducted gene and protein marker analysis allowed us to choose resolution = 0.6 which resulted in 14 distinct clusters. A cluster was removed for having cells with a high percentage of MT counts, and another cluster was also removed due to having only 2 cells, which resulted in a final scRNA data set of 14,681 cells. We isolated and reanalyzed some of these clusters to locate existing subclusters. For each subclustering analysis, we rescaled and repeated dimensional reduction on both integrated RNA and ADT assays of the selected subset of clusters. For each performed subclustering analysis, we built the WNN graph and found existing subclusters that identified 15 subclusters. This workflow generated 22 clusters in total (7 initial clusters and 15 subclusters; annotated in Supplemental Figure 3 and/or Figure 2 [heatmap]). Clusters were defined by manual observation of cluster data. We then applied this workflow on skin and blood samples separately and compared the tissue-specific cluster results to the joint clustering approach.

We used speckle R package incorporating the propeller test for cell-proportion analysis (81). Cytotoxic cells were identified by taking a mean of the normalized expression of *NKG7*, *GZMA*, *GZMB*, *GNLY*, and *PRF1* for each cell. The resulting average cytotoxic expression of cell types was plotted and a cutoff manually selected, yielding 2 distinct distributions (cytotoxic and noncytotoxic).

The AggregateExpression function in the Seurat package was used to aggregate RNA counts from each sample. DESeq and lfcShrink functions from the DESeq2 package (82) were used to conduct the pseudobulk analysis on aggregated counts per sample. Single-cell differential expression analysis on each cluster was performed using likelihood ratio tests from the edgeR package (83, 84) by aggregating raw counts per each cluster of each individual sample. For each comparison, genes with maximum expression across observations of less than 10 counts were removed from analysis. A schematic of scRNA-Seq plus CITE-Seq plus TCR-Seq analysis is shown in Supplemental Figure 15.

### Pathway analysis.

Differentially expressed genes (*P* < 0.05) from the CD8^+^CD103^+^ and CD103^–^ TRM clusters (Supplemental Data Set 2) were analyzed using Ingenuity Pathway Analysis (IPA) software, version 24.0.1 (QIAGEN). Significant enrichment for gene sets in IPA-curated canonical “immune system reactome pathway” and “apoptosis signaling pathway” was determined using Fisher’s exact test *P* values with multiple testing adjustments according to the Benjamini-Hochberg method. Activation or inhibition was determined using *z* score, with *z* score ≥ │2│ considered significant.

### TCR sequence comparison.

We crossreferenced the TCR sequences of all expanded clones from the prospective study dtDHR patients to 2 publicly available databases, VDJdb (42) and McPAS-TCR (43). Searches included T cells with paired α and β chains, or only α or only β chains if the paired chain was not available. We searched against dtDHR patient HLA-A and -B alleles when available in the databases or, if unavailable, against all HLA alleles in the databases. We also compared with published TCR sequences of drug-reactive clones (14, 44–46).

### Mixed lymphocyte reaction, DNA extraction, TCR-β sequencing and HLA typing.

Previously frozen PBMCs from SJS/TEN patient 1 were thawed at 37°C and washed with complete human media (RPMI-1640 plus 4% human AB serum [MilliporeSigma], 1% penicillin/streptomycin, 2 mM l-glutamine, 0.1 mM MEM nonessential amino acids, 10 mM HEPES, 1 mM sodium pyruvate, 50 μM β-mercaptoethanol). Cell pellets were resuspended in complete human media. Cell counts and viability were determined by trypan blue. PBMCs were depleted of CD25^+^ cells using the EasySep Human Pan-CD25 Positive Selection and Depletion Kit (StemCell Technologies) to remove Tregs. CD25^–^ T cells were counted and plated in a 96-well flat-bottom plate in triplicate in complete human media. Cells were incubated with culprit drug bupropion HCL at concentrations of 50 ng/mL and 100 ng/mL, and water for injection (WFI) (Gibco, Thermo Fisher Scientific) as vehicle control. As a positive control, wells were precoated with 500 ng/mL anti-human CD3 (BioLegend) for 2 hours at 37°C, followed by 3 washes with PBS; then cells were plated with 5 μg/mL anti-human CD28. Cells were incubated at 37°C 5% CO_2._ Cells were restimulated with drug or vehicle on day 4. On day 5, cells were collected and washed with PBS. DNA was extracted using the DNeasy Blood and Tissue Kit per the manufacturer’s instructions (QIAGEN). DNA quantity and quality were checked using NanoDrop, then sent for high-throughput TCR-β gene sequencing using immunoSEQ Assay (Adaptive Biotechnologies). DNA was HLA typed by sequence-specific oligonucleotide assay (Luminex LabScan 3D).

### Mice.

HLA-B*57:01 C57Bl/6J transgenic mice were provided by D. Margulies (National Institute of Immunology, Allergy and Infectious Disease, Bethesda, Maryland, USA) and M. Norcross (US Food and Drug Administration, Silver Spring, Maryland, USA). Heterozygous mice were bred inhouse by crossing with C57BL/6J mice (Jackson Laboratory). Male and female mice between 6 and 12 weeks of age were used in experimentation. Mice were phenotyped to confirm transgene expression (59).

HLA-B*57:01^pos^ mice or HLA-B*57:01^neg^ littermate controls were treated by i.p. injection 5 days/week for 17 days with 3 mg of ABC (188062-50-2; Sigma Aldrich) diluted in WFI (59). A subset of mice concurrently underwent treatment of the left ear topically 3 days/week with 0.2 mg ABC in 30% ethanol. Dosing was based on the animal equivalent dosage of ABC (14). Vehicle control mice were treated with equal volume of WFI i.p. injection ± topically with 30% ethanol. All mice were depleted of CD4^+^ T cells by i.p. injection with 0.25 mg anti-CD4 mAb (clone GK1.5, Bio X Cell) in sterile PBS on days –3, +1, +4, and +7 during drug exposure. For in vivo challenge, mice were depleted of CD4^+^ T cells and treated systemically by i.p. injection with ABC or vehicle. Some mice were also treated with FTY720 (1 mg per kg i.p. daily) (SML0700; Sigma-Aldrich).

Mice were monitored thrice weekly for clinical signs of dermatitis and ear thickness measured by electronic digital caliper for the entirety of each study. Mice were harvested at peak of disease (day 22), at disease resolution (day 90), and after drug challenge (day 107).

### Mouse tissue harvest and processing.

Blood was collected in heparin; then PBMCs were isolated by Ficoll gradient. A portion of ear was fixed in 10% neutral buffered formalin, embedded in paraffin, and sectioned at 5 microns for H&E staining by standard method. Spleen, cervical LNs, and remaining ear tissue were harvested into cold PBS. Spleens and LNs were disaggregated over 70 μm strainers into single-cell suspensions. Ear skin was cut into small pieces, then incubated in 3 mL HBSS (14175095; Thermo Fisher Scientific) with 1 mg/mL collagenase A (11088785103; Roche) and 40 μg/mL DNase I (10104159001; Roche) at 37°C for 3 hours in a shaking incubator. To neutralize the collagenase, RPMI plus 10%FBS was added to the tubes and suspension centrifuged (465*g*, 5 minutes, 4°C). Cell pellets were resuspended in complete RPMI media then disaggregated through a 70 μm filter. Cell counts and viability were assessed with trypan blue.

### Mouse flow cytometry.

Single-cell suspensions of ear skin, blood, cervical LNs, and spleen were blocked with 5% normal goat serum. Samples were surface stained with combinations of the following: PE/Cy7-CD3 (17A2), PERCP-CD8a (53-6.7), APC/Cy7-CD8a (53-6.7), FITC-CD44 (IM7), PE-CD69 (H1.2F3), and APC-CD62L (MEL-14). CLA expression was assessed by incubating cells with E-selectin/Fc chimera (575-ES; R&D Systems) in conjunction with PerCP-conjugated F(ab′)2 fragments of goat anti-human IgG F(c) antibody (109-126-170; Jackson ImmunoResearch). For intracellular cytokine staining, cells were fixed and permeabilized using the Cytofix/Cytoperm Fixation/Permeablization Kit (554714; BD Biosciences) according to the manufacturer’s instructions and stained with FITC–IFN-γ (XMG1.2), PE–TNF-α (MP6-XT22), and/or APC–granzyme B (QA16A02) (BioLegend).

### Statistics: lymphopenic human and mouse studies.

Two groups were compared using a 2-tailed Mann-Whitney *U* test in Graph Pad Prism (version 9). Three groups were compared with a Kruskal-Wallis test, and if significant, subsequently by Dunn’s test for multiple comparisons. *P* < 0.05 was considered statistically significant.

### Study approval.

Approval for human studies was granted by the Partners Healthcare IRB (no. 2002P001345, no. 2014P000615, no. 2016P001357, no. 2017P002826, no. 2018P001497) and BCH IRB-P00024905. Written consent was obtained prior to participation in the prospective study. Mouse experiments were performed in accordance with the guidelines put forth by the Center for Animal Resources and Comparative Medicine at Harvard Medical School (IACUC approval 2016N000070).

### Data availability.

Values for all data points in graphs are reported in the Supporting Data Values file. Data are available in the dbGaP public repository (accession number phs003344.v1.p1) and/or upon request. Only deidentified human data will be shared. Supporting analytic code can be accessed at https://github.com/garber-lab/ShahSJSanalysis (commit ID a2105a5).

## Author contributions

SJD, PNS, GAR, and KW designed the study. SJD, PNS, GAR, PCH, XF, and RTB planned and executed experiments and/or data acquisition. SJD, GAR, PNS, GAV, EMS, PCH, IG, AHW, A Mostaghimi, FRV, MGL, BARS, and RKF provided patient sample collection, processing, and/or medical record data collection. AGH, VB, A Manukyan, WCK, and MG performed statistical analysis and/or mathematical modeling. SJD, PNS, GAR, VB, A Manukyan, WCK, and MG prepared the manuscript. PNS, GAR, A Manukyan, and WCK contributed substantially to this work, warranting co–first authorship. Relative contributions are reflected in authorship order.

## Supplementary Material

Supplemental data

Supplemental data set 1

Supplemental data set 2

Supplemental data set 3

Supporting data values

## Version 1 07/23/2024

In-Press Preview

## Version 2 09/03/2024

Electronic publication
