# Supplementary material for: Systemic and skin-limited delayed-type drug hypersensitivity reactions associate with distinct resident and recruited T cell subsets
Source: J Clin Invest. 2024 Jul 23;134(17):e178253. doi: 10.1172/JCI178253 (PMC11364394; doi:10.1172/JCI178253)
Supplement: Supplemental data [file jci-134-178253-s212.pdf]

Supplemental Material

Supplemental Table 1. Fold changes and adjusted p-values for dtDHR vs healthy skin by bulk transcriptional profiling.

	SJS/TEN		DRESS		MDE	
Genes	Log_2FC	P_{adj}	Log_2FC	P_{adj}	Log_2FC	P_{adj}
ABCF1	0.37	0.00044	0.18	0.23884	0.34	0.02497
AHR	-0.67	4.35E-09	-0.86	2.36E-09	-0.69	7.52E-06
ALAS1	0.71	0.00532	0.32	0.37049	0.22	0.59649
ARG1	0.07	0.91347	-0.43	0.56904	-0.23	0.81697
B2M	**1.39**	**2.35E-11**	0.78	0.00576	0.96	0.00092
CASP1	**1.33**	**1.27E-07**	0.38	0.31163	0.44	0.25391
CASP10	**1.15**	**2.02E-08**	0.47	0.12147	0.51	0.09958
CASP2	-0.21	0.11885	0.08	0.6409	-0.16	0.40135
CASP3	0.37	0.01393	0.24	0.24864	0.01	0.9674
CASP8	0.31	0.17328	0.56	0.05058	0.11	0.7812
CCL11	-0.7	0.23626	-0.7	0.34956	-0.26	0.80918
CCL13	**1.15**	**0.00187**	**2.64**	**3.26E-09**	**2.19**	**5.07E-06**
CCL16	-0.46	0.39357	-0.64	0.35612	-1.01	0.15695
CCL18	1.17	0.05954	**3.55**	**1.40E-06**	**3.26**	**3.05E-05**
CCL2	**2.23**	**6.67E-06**	0.78	0.29443	0.97	0.18967
CCL20	**1.06**	**0.04466**	-0.41	0.53001	-0.38	0.61761
CCL22	0.07	0.85219	0.93	0.07381	0.29	0.65991
CCL23	0.63	0.15642	**1.62**	**0.0017**	0.8	0.18774
CCL24	**-1.15**	**0.02584**	0.19	0.74875	-1.28	0.06318
CCL26	-0.69	0.19748	**1.46**	**0.02095**	0.82	0.24308
CCL27	**-1.28**	**0.01275**	-0.03	0.97711	0.59	0.39491
CCL5	**2.56**	**4.97E-10**	**3.29**	**1.51E-10**	**2.08**	**0.00019**
CCL7	**2.67**	**9.57E-06**	-0.29	0.64728	-0.54	0.47286
CCL8	**3.82**	**6.74E-13**	0.81	0.32491	0.81	0.36449
CCR1	**1.87**	**4.12E-08**	**1.06**	**0.02487**	0.85	0.10218
CCR10	-0.52	0.31476	-0.7	0.29366	-0.95	0.15695
CCR2	**1.47**	**2.84E-05**	**1.34**	**0.0035**	0.59	0.28214
CCR4	-0.52	0.1842	0.05	0.91423	-0.78	0.13454
CCR5	**2.31**	**2.60E-09**	**2.51**	**2.13E-07**	**1.71**	**0.00146**
CCR6	-0.59	0.16193	-0.94	0.07618	-0.91	0.10215
CCR7	**1.05**	**0.0042**	**1.05**	**0.02727**	0.32	0.61356
CD14	**1.94**	**6.27E-10**	**1.34**	**0.00134**	**1.08**	**0.01968**
CD1A	**-2.04**	**2.02E-09**	**-1.08**	**0.02198**	-0.92	0.0746
CD1D	-0.14	0.76198	-0.47	0.42263	-0.53	0.39491
CD209	**1.64**	**2.06E-06**	**2.37**	**4.06E-08**	**1.89**	**3.81E-05**

CD244	-0.26	0.64182	-0.42	0.56074	-0.77	0.29908
CD27	0.64	0.13446	**1.05**	**0.0483**	0.32	0.6481
CD274	**2.86**	**9.98E-09**	1.24	0.08623	1.07	0.16226
CD276	0.45	0.04954	0.27	0.39301	0.22	0.53892
CD28	0.2	0.58633	0.76	0.08704	-0.12	0.83123
CD3D	0.72	0.05186	**1.4**	**0.00169**	0.37	0.51987
CD3E	**1.04**	**0.0016**	**1.86**	**2.86E-06**	0.92	0.05338
CD4	0.82	0.00109	0.98	0.00225	0.64	0.0793
CD40	**1.62**	**3.58E-07**	**1.04**	**0.01832**	0.91	0.05483
CD40LG	-0.87	0.01895	-0.04	0.95796	-0.41	0.46475
CD44	0.14	0.4412	0.41	0.08623	**0.5**	**0.03977**
CD45R0	**1.76**	**1.27E-08**	**1.35**	**0.00107**	**1.05**	**0.02113**
CD45RA	**1.28**	**0.00082**	0.89	0.09254	0.44	0.47286
CD69	-0.35	0.41903	-0.45	0.42263	**-1.18**	**0.03049**
CD7	**2.24**	**4.06E-09**	**2.31**	**1.32E-06**	**1.16**	**0.04251**
CD80	0.97	0.04263	0.6	0.34573	0.24	0.7812
CD86	**1.24**	**0.00038**	0.33	0.52397	0.09	0.90695
CD8A	**1.5**	**0.00053**	**2.17**	**3.94E-05**	0.86	0.18774
CD8B	0.04	0.91989	0.57	0.31665	-0.42	0.49368
CSF1	0.65	0.0094	0.05	0.90964	0.02	0.9674
CSF1R	0.84	0.00738	0.68	0.11767	0.3	0.55045
CSF2	-0.51	0.32133	-0.42	0.52874	-0.51	0.48421
CTLA4_all	0.91	0.01926	0.81	0.12147	-0.15	0.79948
CTLA4-TM	**1.93**	**9.30E-09**	**1.93**	**4.54E-06**	0.86	0.09907
CX3CL1	**1.12**	**0.0061**	-0.14	0.80613	0.1	0.90259
CX3CR1	0.03	0.93785	-0.04	0.95796	-0.15	0.81697
CXCL1	**2.24**	**1.47E-06**	**1.92**	**0.00156**	**1.69**	**0.0107**
CXCL10	**6.08**	**4.57E-25**	**3.74**	**3.09E-07**	**4.4**	**2.83E-09**
CXCL11	**4.87**	**1.18E-16**	**2.36**	**0.00224**	**2.79**	**0.00037**
CXCL2	0.54	0.23289	-0.1	0.88672	-0.7	0.23087
CXCL9	**5.16**	**1.62E-26**	**4.42**	**9.29E-13**	**4.14**	**4.13E-11**
CXCR3	0.48	0.31476	0.98	0.09254	0.03	0.98174
CXCR4	-0.37	0.3531	-0.05	0.95796	**-1.14**	**0.0233**
EEF1G	**-1.06**	**4.16E-10**	-0.77	0.00068	**-0.63**	**0.01067**
FADD	0.19	0.41993	0.24	0.43138	0.04	0.92515
FAS	-0.24	0.21427	-0.39	0.12707	-0.35	0.18774
FASLG	0.83	0.07712	0.92	0.12739	0.14	0.88671
FOXP3	-0.68	0.1764	-0.93	0.14349	-0.96	0.15695
G6PD	0.34	0.13446	0.03	0.95546	0.3	0.35699
GAPDH	0.34	0.0065	0.65	2.32E-05	0.32	0.07553
GATA3	**-2.07**	**7.18E-13**	**-1.19**	**0.0019**	-0.55	0.217
GNLY	**2.1**	**4.99E-06**	**2.44**	**1.81E-05**	0.7	0.3657
GUSB	-0.24	0.14718	-0.25	0.25504	-0.41	0.06318
GZMA	**2.53**	**2.02E-08**	**3.4**	**1.41E-09**	**1.9**	**0.00271**

GZMB	**3.52**	**2.93E-13**	**3.14**	**1.66E-07**	**2.06**	**0.00231**
GZMK	0.67	0.13446	**1.87**	**0.00028**	0.34	0.65258
HLA-DRA	**1.31**	**4.99E-06**	**1.11**	**0.00361**	0.93	0.02706
HLA-DRB1	**1.52**	**0.00773**	0.89	0.25504	0.72	0.39491
HLA-DRB3	**1.87**	**4.90E-14**	**1.75**	**3.17E-08**	**1.55**	**5.07E-06**
HLA-E	**1.08**	**3.77E-08**	0.69	0.00999	0.63	0.03049
HOBIT	0.78	0.17116	1.28	0.06735	0.22	0.84621
HPRT1	0.07	0.6427	0.06	0.7705	-0.07	0.77838
ICAM1	**1.78**	**7.47E-07**	0.96	0.06098	0.7	0.2045
ICAM3	0.58	0.01584	0.83	0.00636	0.04	0.93891
ICOS	0.33	0.49333	0.44	0.46214	-0.4	0.53892
ICOSLG	-0.2	0.47955	-0.47	0.21011	-0.34	0.39491
IDO1	**4.9**	**5.34E-16**	**1.77**	**0.03974**	**2.43**	**0.00318**
IFNA2	-0.57	0.37334	-1.18	0.13209	-1.17	0.15695
IFNG	0.85	0.13461	-0.02	0.97016	-0.64	0.37218
IFNGR1	-0.07	0.62885	-0.08	0.65189	-0.08	0.73077
IL10	**2.18**	**1.16E-13**	**2.2**	**1.86E-09**	**1.62**	**3.81E-05**
IL10RA	**1.4**	**6.25E-08**	**1.15**	**0.00085**	0.63	0.10892
IL12A	0.59	0.03904	0.09	0.85687	-0.08	
IL12B	-0.2	0.71122	-0.48	0.50051	-0.87	0.217
IL12RB1	**1**	**0.01823**	0.91	0.10681	0.22	0.7812
IL13	-0.24	0.62591	-0.13	0.87424	-0.25	0.75271
IL13RA1	0.19	0.24394	0.29	0.17223	0.1	0.69168
IL15	0.61	0.05186	0.09	0.85687	0.02	0.9674
IL16	-0.39	0.14336	-0.17	0.63734	-0.84	0.01355
IL17B	-0.39	0.41579	-0.8	0.19572	-1.04	
IL17F	-0.74	0.23219	-1.16	0.13209	-1.09	0.18256
IL18	-0.42	0.19224	**-1.02**	**0.00951**	-0.6	0.17757
IL18R1	0.19	0.44589	0.49	0.12147	-0.44	0.18774
IL18RAP	0.61	0.14718	0.48	0.37407	-0.08	0.90332
IL1A	0.67	0.17018	-0.44	0.46214	-0.64	0.31628
IL1B	2.1	0.29502	-0.38	0.53866	-0.57	0.39491
IL1R1	0.31	0.08775	0.07	0.82098	0.12	0.69168
IL1R2	**1.47**	**0.00041**	**1.21**	**0.02727**	0.16	0.87036
IL1RAP	-0.15	0.46243	-0.4	0.12739	-0.25	0.37768
IL1RN	**2.06**	**7.03E-10**	0.45	0.37049	0.25	0.69168
IL2	-0.7	0.22115	-0.94	0.19572	-1.21	0.10218
IL20	-0.34	0.54514	-0.52	0.45454	-0.46	0.57255
IL21	-0.25	0.6427	-0.27	0.74073	-0.67	0.37768
IL21R	0.58	0.17825	0.56	0.31364	-0.1	0.87316
IL22	-0.06	0.90813	-0.81	0.24908	-0.71	0.35632
IL22RA2	0.08	0.79164	0.11	0.83983	0.29	
IL23A	0.15	0.76362	-0.38	0.53001	-0.72	0.24546
IL23R	-0.55	0.3531	-0.88	0.238	-1.08	0.15695

IL27	-0.05	0.92253	-0.84	0.24908	-1.22	0.0963
IL2RA	0.68	0.07539	0.74	0.13656	0.09	0.90623
IL2RB	**1.22**	**0.00078**	**1.62**	**0.00036**	0.59	0.28747
IL2RG	**1.38**	**3.62E-06**	**1.73**	**3.30E-06**	0.86	0.05451
IL32	**1.77**	**2.33E-08**	**2.03**	**3.84E-07**	**1.28**	**0.00446**
IL4	0.1	0.84699	-0.46	0.46214	-0.6	0.3657
IL4R	**1.52**	**6.74E-13**	**1.44**	**8.99E-08**	**1.17**	**3.81E-05**
IL5	-0.53	0.39621	-0.76	0.34349	-1.01	0.20744
IL6	0.19	0.78598	-1.37	0.07174	-1.39	0.0793
IL6R	0.68	0.00201	0.33	0.2887	-0.07	0.86651
IL6ST	-0.08	0.61889	-0.42	0.02095	-0.42	0.03043
IL7	-0.26	0.53046	-0.66	0.19634	-0.56	0.30684
IL7R	0.37	0.2676	0.8	0.0565	-0.21	0.67804
IL8	**2.78**	**5.02E-06**	1.2	0.16186	0.5	0.69168
IL9	-0.56	0.29405	-0.32	0.6409	-0.25	0.7812
ITGAE	0.44	0.38169	-0.14	0.85687	-0.85	
ITGAL	**2.11**	**1.26E-10**	**2.26**	**4.06E-08**	**1.51**	**0.00092**
ITGAM	**1.5**	**3.64E-12**	**1.26**	**4.90E-06**	0.9	0.00318
ITGAX	**1.84**	**3.05E-10**	**1.26**	**0.00116**	**1.05**	**0.01352**
ITGB2	**2.14**	**1.24E-18**	**2.17**	**3.02E-12**	**1.45**	**1.23E-05**
JAK1	-0.04	0.7744	-0.03	0.88917	-0.15	0.51031
JAK2	**1.07**	**0.00024**	0.29	0.49169	0.48	0.27762
JAK3	**1.93**	**1.04E-12**	**2.39**	**3.02E-12**	**1.51**	**3.81E-05**
KIR_Activating_Subgroup_1	-0.35	0.6059	-1.11	0.17451	-1.17	0.16967
KIR_Activating_Subgroup_2	-0.74	0.25829	-1.1	0.19057	-1.57	0.05483
KIR_Inhibiting_Subgroup_1	-0.33	0.62591	-1.01	0.24908	-1.24	0.15695
KIR_Inhibiting_Subgroup_2	**2.14**	**3.77E-08**	0.87	0.12739	0.96	
KLRAP1	-0.32	0.07035	-0.45	0.04813	0.03	0.92515
KLRB1	-0.53	0.18372	-0.58	0.26219	-0.33	0.5942
KLRC1	0.79	0.11076	0.11	0.89295	-0.23	0.75271
KLRC4	-0.17	0.73979	-0.56	0.4018	-0.64	0.3657
KLRD1	**1.45**	**1.18E-05**	**1.95**	**1.84E-06**	0.79	
KLRF1	-0.34	0.47955	-0.79	0.19572	-0.97	0.12301
KLRF2	-0.44	0.46641	-0.7	0.35854	-1.05	0.17757
KLRG1	-0.77	0.03965	-0.79	0.11613	-0.8	0.12599
KLRG2	-0.44	0.3706	-0.62	0.3232	-0.84	0.18967
LAMP1	-0.24	0.07539	-0.52	0.00134	-0.13	0.51031
MICA	0.25	0.23219	0.01	0.97672	-0.17	0.59649
MICB	0.61	0.05186	0.21	0.63734	0.02	0.9674
NLRP3	0.15	0.62591	-0.23	0.55677	-0.31	0.47813
NOS2	-0.31	0.6059	-0.95	0.20324	-1.45	0.05017
OAZ1	0.48	0.00059	0.22	0.27318	0.25	0.217
PDCD1	-0.02	0.96186	-0.43	0.5434	-0.89	0.20471
PDCD1LG2	**1.62**	**3.58E-07**	**1**	**0.02198**	0.87	0.06593

PDCD2	0.52	0.01577	0.38	0.19572	0.33	0.28838
PECAM1	-0.11	0.6427	-0.32	0.31665	-0.55	0.08864
POLR1B	0.1	0.62591	-0.19	0.49301	0.07	0.84621
POLR2A	-0.1	0.37428	-0.12	0.42113	0.01	0.93891
PRF1	**2.33**	**2.60E-09**	**2.34**	**1.84E-06**	**1.17**	**0.04878**
S1PR1	-0.14	0.60804	-0.35	0.3232	-0.64	0.07553
sCTLA4	-0.11	0.54454	0.01	0.95796	-0.13	
SELE	0.98	0.05024	-0.31	0.61062	0.05	0.9674
SELL	**1.89**	**4.87E-06**	**1.66**	**0.0019**	0.71	0.27762
SELPLG	0.29	0.47955	0.29	0.58015	-0.26	0.67804
STAT1	**3.1**	**2.59E-30**	**2.3**	**4.28E-11**	**2.24**	**3.10E-10**
STAT2	**2.03**	**2.59E-16**	**1.19**	**0.00028**	**1.34**	**5.67E-05**
STAT3	0.81	2.40E-07	0.58	0.00636	0.7	0.00141
STAT4	0.66	0.02693	0.67	0.09254	-0.02	0.94982
STAT5A	0.68	0.00038	0.57	0.02841	0.5	0.07567
STAT5B	-0.24	0.05736	-0.23	0.17451	-0.35	0.04156
STAT6	0.47	2.46E-06	0.32	0.02198	0.32	0.03043
TBP	-0.5	0.00251	-0.55	0.01158	-0.55	0.01686
TBX21	0.46	0.35639	0.46	0.45454	-0.17	0.81697
TGFB1	**1.19**	**6.86E-13**	**1.03**	**1.09E-06**	0.65	0.00662
TGFBR1	0.46	0.00389	0.22	0.32491	0.03	0.92732
TGFBR2	0.47	0.03978	-0.02	0.95796	-0.07	0.86651
TNF	**1.15**	**0.00172**	0.39	0.45454	0.27	0.67689
TNFRSF1B	**1.81**	**6.65E-19**	**1.44**	**3.17E-08**	0.92	0.00142
TNFRSF4	-0.5	0.369	-1.05	0.12739	-1.16	0.10218
TNFRSF9	0.59	0.18988	0.1	0.88672	-0.12	0.86651
TNFSF10	**1.8**	**4.35E-09**	0.96	0.02459	**1**	**0.02706**
TNFSF11	**-1.17**	**0.01344**	-0.69	0.29512	-0.67	
TNFSF4	-0.4	0.37535	-0.84	0.13523	-0.91	0.12599
TUBB	0.21	0.18372	0.29	0.16043	0.27	0.21303
TYROBP	**2.51**	**4.46E-23**	**2.03**	**5.71E-10**	**1.55**	**8.77E-06**
VCAM1	0.82	0.01321	0.75	0.09268	0.71	0.13247

Significance = P_{adj} <0.05 and $|Log_2FC| \geq 1$

Supplemental Table 2. Fold changes and adjusted p-values for SJS/TEN vs MDE and SJS/TEN vs DRESS by bulk transcriptional profiling; showing genes with $P_{adj} < 0.1$ and $|Log_2FC| \geq 1$.

	SJS/TEN vs. MDE	
**Genes**	Log_2FC	P_{adj}
CCL8	3.01	8.45E-05
CCL7	3.20	8.45E-05
IL1RN	1.81	1.21E-04
GATA3	-1.52	2.09E-04
CCL27	-1.88	0.016
IDO1	2.48	0.018
IL8	2.28	0.018
CCL18	-2.08	0.031
CD274	1.79	0.031
CD86	1.16	0.038
IL1R2	1.31	0.046
CXCL11	2.09	0.046
CD1A	-1.12	0.046
CTLA4-TM (membrane-bound form)	1.08	0.047
CCL26	-1.51	0.047
PRF1	1.16	0.052
CTLA4_all (common probe)	1.06	0.052
IFNG	1.49	0.052
IL1A	1.31	0.052
CCL13	-1.04	0.052
CCR1	1.03	0.052
CCL20	1.44	0.052
ITGAE	1.29	0.052
ICAM1	1.08	0.052
GZMB	1.46	0.052
KIR_Inhibiting_Subgroup_2	1.18	0.052
GNLY	1.40	0.052
CXCL2	1.24	0.052
IL6	1.58	0.054
SELL	1.18	0.056
CD7	1.08	0.061
CX3CL1	1.01	0.086
IL1B	2.67	0.096
CCL2	1.26	0.096

	SJS/TEN vs. DRESS	
**Genes**	Log_2FC	P_{adj}
CCL8	3.01	8.52E-05
CCL7	2.96	3.58E-04
IDO1	3.14	3.58E-04
IL1RN	1.61	8.77E-04
CCL26	-2.15	0.001
CCL13	-1.49	0.004
CCL18	-2.37	0.006
CXCL11	2.52	0.006
CXCL10	2.34	0.020
CX3CL1	1.26	0.044
CD274	1.62	0.050
GZMK	-1.20	0.061
CCL20	1.47	0.061
CCL2	1.45	0.078
SELE	1.29	0.079
IL6	1.56	0.079

There were no differentially expressed genes between DRESS and MDE with $P_{adj} < 0.1$ and $|Log_2FC| \geq 1$.

Supplemental Table 3. Prospective study patient demographic and clinical data

dtDHR (severity of rash if MDE)	Age/ Sex	Race/ Ethnicity[a]	HLA-A & HLA-B	Suspected culprit drug(s)	Infection testing/results	Histopathologic description	Immunosuppressive treatment prior to study sampling[b]
SJS/TEN 1	45M	Black or African-American/ Non-hispanic	HLA-A: 03:01 32:01 HLA-B: 44:02 44:03	Bupropion	Mycoplasma negative	Lymphocyte-mediated interface dermatitis	No
SJS/TEN 2	47M	NA[c]	HLA-A: 02:01 02:05 HLA-B: 44:02 49:01	Sulfamethoxazole /trimethoprim	None	Full-thickness epidermal necrosis and mild superficial perivascular lymphocytic infiltrate	Systemic steroids
SJS/TEN 3	37F	White/ Non-hispanic	HLA-A: 03:01 32:01 HLA-B: 44:02 51:05	Ceftaroline, Gentamycin	Mycoplasma negative HSV negative HBV negative HIV negative	Lymphocyte-mediated interface dermatitis	No
MDE 1 (mild)	50M	White/ Non-hispanic	HLA-A: 03:01 68:01 HLA-B: 44:02 44:03	Oxacillin	HAV negative HBV negative HCV negative HIV negative	Focal interface dermatitis with marked dermal edema and scattered interstitial lymphocytes, neutrophils and eosinophils	Topical steroid
MDE 2 (severe)	56M	White/ Non-hispanic	HLA-A: 02:01 33:03 HLA-B: 44:02 50:01	Allopurinol	Mycoplasma negative CMV negative EBV negative	Spongiotic dermatitis with pityriasiform scale, superficial perivascular lymphocytic infiltrate, and eosinophils	Systemic steroids
MDE 3 (mild)	75F	White/ Non-hispanic	HLA-A: 02:01 29:01/ 29:02 HLA-B: 44:01 44:02	Ceftazidime	None	Spongiotic dermatitis with hyperkeratosis and acanthosis, with perivascular and interstitial lymphohistiocytic infiltrate	Topical steroid

[a]Race/Ethnicity as patient-reported in the medical record
[b]Immunosuppressive treatment (one or more doses) administered for dtDHR prior to study sample collection
[c]Not available

Supplemental Table 4. Marker list used to define T cell clusters in scRNAseq + CITEseq + TCRseq.

Cluster number	Cluster name	Markers
1	CD8^{+} Naïve	CD45RA^{+} CD45RO^{-} IL7Rα^{high} *S1PR1*$^{+}$ *KLF2*$^{+}$ *CCR7*$^{+}$
2	CD8^{+} TCM	CD45RA^{-} CD45RO^{+} IL7Rα^{high} *S1PR1*$^{+}$ *KLF2*$^{+}$ CD62L^{high}
3	CD8^{+} TMM (migratory memory) *(1)*	CD45RA^{-} IL7Rα^{high} *S1PR1*^{low} *KLF2*$^{+}$ CD62L^{low} *CCR7*$^{+}$
4	CD8^{+} CD103^{-} TRM (non-functional)	CD45RA^{-} CD45RO^{+} IL7Rα^{high} *S1PR1*$^{-}$ *KLF2*$^{-}$ CD62L^{low} *CCR7*$^{-}$ CD103^{-} CD69^{+} *CX3CR1*$^{-}$<br>Not cytotoxic*
5	CD8^{+} CD103^{-} TEMRA	CD45RA^{+} IL7Rα^{low} CD62L^{low} *CCR7*$^{-}$ CD103^{-} CD69^{-} *CX3CR1*$^{+}$
6	CD8^{+} CD103^{+} TEMRA	CD45RA^{+} IL7Rα^{low} CD62L^{low} *CCR7*$^{-}$ CD103^{+} CD69^{-} *CX3CR1*$^{+}$
7	CD8^{+} T effectors	CD45RO^{-} IL7Rα^{low} *S1PR1*$^{-}$ *KLF2*$^{-}$ CD62L^{low} *CCR7*$^{-}$ *CX3CR1*$^{+}$
8	CD8^{+} TEM	CD45RA^{-} CD45RO^{+} IL7Rα^{low} *S1PR1*$^{-}$ *KLF2*$^{-}$ CD62L^{low} *CCR7*$^{-}$ CD69^{-} *CX3CR1*$^{+}$
9	CD8^{+} CD103^{+} TRM	CD45RA^{-} IL7Rα^{low} *S1PR1*$^{-}$ *KLF2*$^{-}$ CD62L^{low} *CCR7*$^{-}$ CD103^{+} CD69^{+} *CX3CR1*$^{-}$<br>Cytotoxic*
10	CD8^{+} CD103^{-} TRM	CD45RA^{-} IL7Rα^{low} *S1PR1*$^{-}$ *KLF2*$^{-}$ CD62L^{low} *CCR7*$^{-}$ CD103^{-} CD69^{+} *CX3CR1*$^{-}$<br>Cytotoxic*
11	CD8^{+} CD56^{+} T cells	CD45RA^{+} CD45RO^{-} IL7Rα^{low} CD62L^{low} *CCR7*$^{-}$ CD56^{+}
12	γδ T cells	CD4^{-} CD8^{-} *TRDV2*$^{+}$
13	CD4^{+} Naïve	CD45RA^{+} CD45RO^{-} IL7Rα^{med} *S1PR1*$^{+}$ *KLF2*$^{+}$ CD62L^{high} *CCR7*^{high}
14	CD4^{+} CD45RA^{+} CD62L^{low}	CD45RA^{+} CD45RO^{-} IL7Rα^{med} *S1PR1*$^{+}$ *KLF2*$^{+}$ CD62L^{low} *CCR7*$^{+}$
15	CD4^{+} CD45RA^{-} CD62L^{low}	CD45RA^{-} CD45RO^{-} IL7Rα^{med} *S1PR1*$^{+}$ *KLF2*$^{+}$ CD62L^{low} *CCR7*^{low}
16	CD4^{+} TCM	CD45RA^{-} CD45RO^{+} IL7Rα^{high}*S1PR1*$^{+}$ *KLF2*$^{+}$ CD62L^{med}
17	CD4^{+} CD103^{-} TRM	CD45RA^{-} CD45RO^{+} IL7Rα^{high}*S1PR1*$^{-}$ *KLF2*$^{-}$ CD62L^{low} *CCR7*$^{-}$ CD103^{-} CD69^{+}
18	CD4^{+} CD103^{+} TRM	CD45RA^{-} CD45RO^{+} IL7Rα^{high} *S1PR1*$^{-}$ *KLF2*$^{-}$ CD62L^{low} *CCR7*$^{-}$ CD103^{+} CD69^{+}
19	CD4^{+} T effectors	IL7Rα^{low} *S1PR1*$^{-}$ CD62L^{low} *CCR7*$^{-}$ *CX3CR1*$^{+}$
20	CD4^{+} Treg 1	CD69^{+} FAS^{+} *FOXP3*$^{+}$
21	CD4^{+} Treg 2	FAS^{+} *FOXP3*$^{+}$ *CTLA4*$^{+}$ *IL2RA*$^{+}$
22	Proliferating	*TUBA1B*$^{+}$ *STMN1*$^{+}$

Gene name is italicized if RNA was used; protein name is not italicized if protein was used.
*Cytotoxic defined as expressing a combination of *GNLY*$^{+}$ *GZMA*$^{+}$ *GZMB*$^{+}$ *PRF1*$^{+}$and/or *NKG7*$^{+}$ based on the heatmap.

Supplemental Table 5. Clinical and histopathologic characteristics of lymphopenic AML patients with MDE.

Age/ Sex	Chemotherapy	No. days after chemotherapy that skin was biopsied	Day of Biopsy		Histopathologic description
			WBC (K/μl)	Absolute lymphocyte count (K/μl)[b]	
48M	Amonafide + Daunorubicin	20	0.12	0.166	Mild superficial and deep perivascular predominantly lymphoid infiltrate with scattered eosinophils
67M	Cytarabine + Daunorubicin + Bortezomib	16	0.19	0.109	Superficial and mid-perivascular predominantly lymphoid infiltrate
54F	Cytarabine	14	0.10	0.10	Spongiosis with superficial perivascular chronic inflammation[a] with dermal hemorrhage
44M	Mitoxantrone + Etoposide + Cytarabine	6	0.2	0.12	Lymphocyte-mediated interface dermatitis
56F	Mitoxantrone + Etoposide	32	0.11	0.103	Mixed lymphocyte-mediated interface and spongiotic dermatitis
64F	Mitoxantrone + Etoposide + Cytarabine	42	0.33	0.05	Focal vacuolar interface changes with perivascular chronic inflammation[a] with eosinophils
62M	Cytarabine + Daunorubicin	8	0.52	0.468	Spongiotic and interface dermatitis with perivascular lymphocytic infiltrate
65F	Cytarabine + Daunorubicin	23	0.97	0.165	Spongiosis with lymphocytic exocytosis, focal minimal interface change, superficial perivascular chronic inflammation
45M	Etoposide + Cytarabine + Daunorubicin	6	0.42	0.244	Perivascular chronic inflammation[a] with eosinophils
59M	Cytarabine + Idarubicin	17	0.57	0.542	Superficial dermal edema with perivascular chronic inflammation[a]
70M	Amonafide + Cytarabine	38	0.45	0.432	Superficial mid-dermal infiltrate with lymphoid and plasma cells
57M	Daunorubicin + Cytarabine	10	0.13	0.095	Subacute spongiotic dermatitis

[a] "Chronic inflammation" on pathology refers to either lymphocytic or lymphohistocytic infiltrate
[b] Absolute lymphocyte count is reported from the day of biopsy, or day after biopsy if differential was not obtained on the day of biopsy.

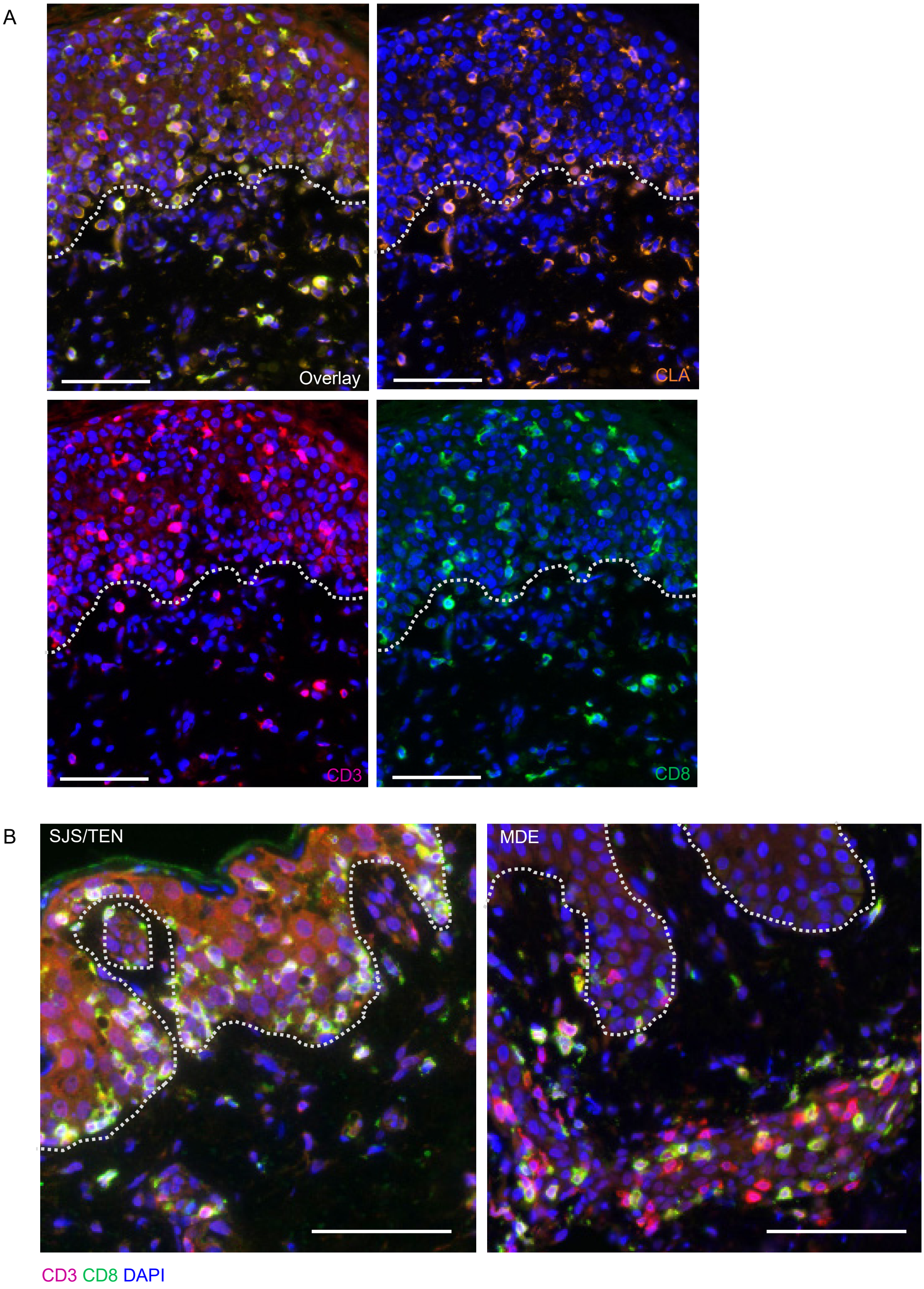

Supplemental Figure 1

Supplemental Figure 1. Visualization of T cell infiltrate in dtDHR skin samples. (A) Representative immunofluorescence images of CD3 (magenta), CD8 (green) and CLA (orange) with overlay, of an early (non-blistered) SJS/TEN sample showing a predominantly epidermal $CD8^+$ skin homing (CLA^+) T cell infiltrate. **(B)** Representative immunofluorescence overlay images of CD3 (magenta) and CD8 (green) staining showing a $CD8^+$ T cell infiltrate predominantly in the epidermis in SJS/TEN compared to a mixed $CD8^+$ and $CD8^-$ T cell infiltrate predominantly around vessels and in the papillary dermis in MDE. **(A,B)** Gray dotted line depicts dermoepidermal junction. White lines = 100 μm.

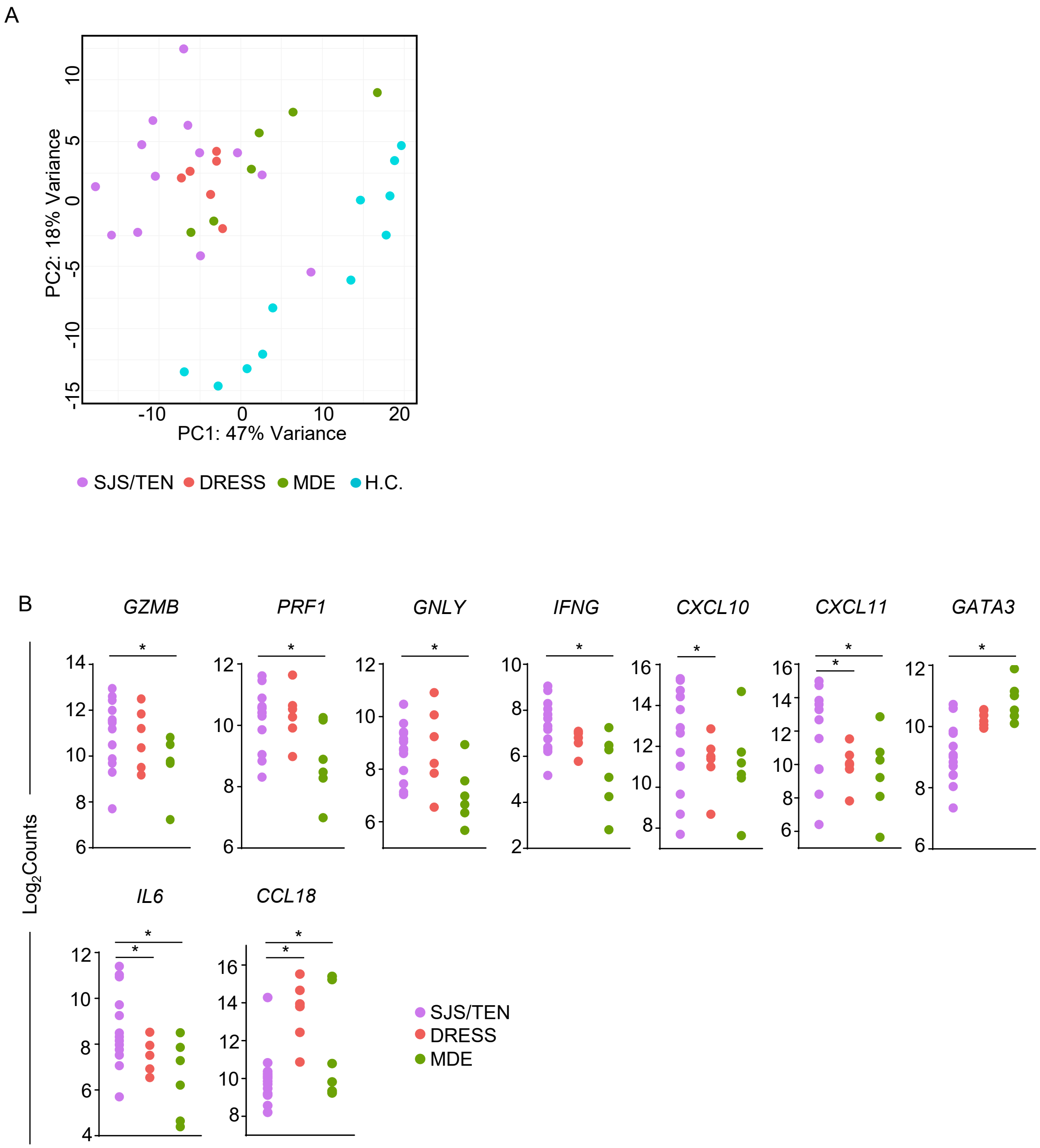

Supplemental Figure 2

Supplemental Figure 2. Bulk transcriptional profiling with differential gene expression analysis between dtDHR. **(A)** PCA plot from bulk transcriptional profiling of SJS/TEN (N=13), DRESS (N=6), MDE (N=6) and healthy control (H.C.) (N=11). **(B)** Log2counts of genes relevant for T cell functionality and/or Treg recruitment and development in SJS/TEN compared to DRESS and MDE. *Significance defined as $|Log_2FC| \geq 1$ and $P_{adj} < 0.1$, DESeq2, Wald test

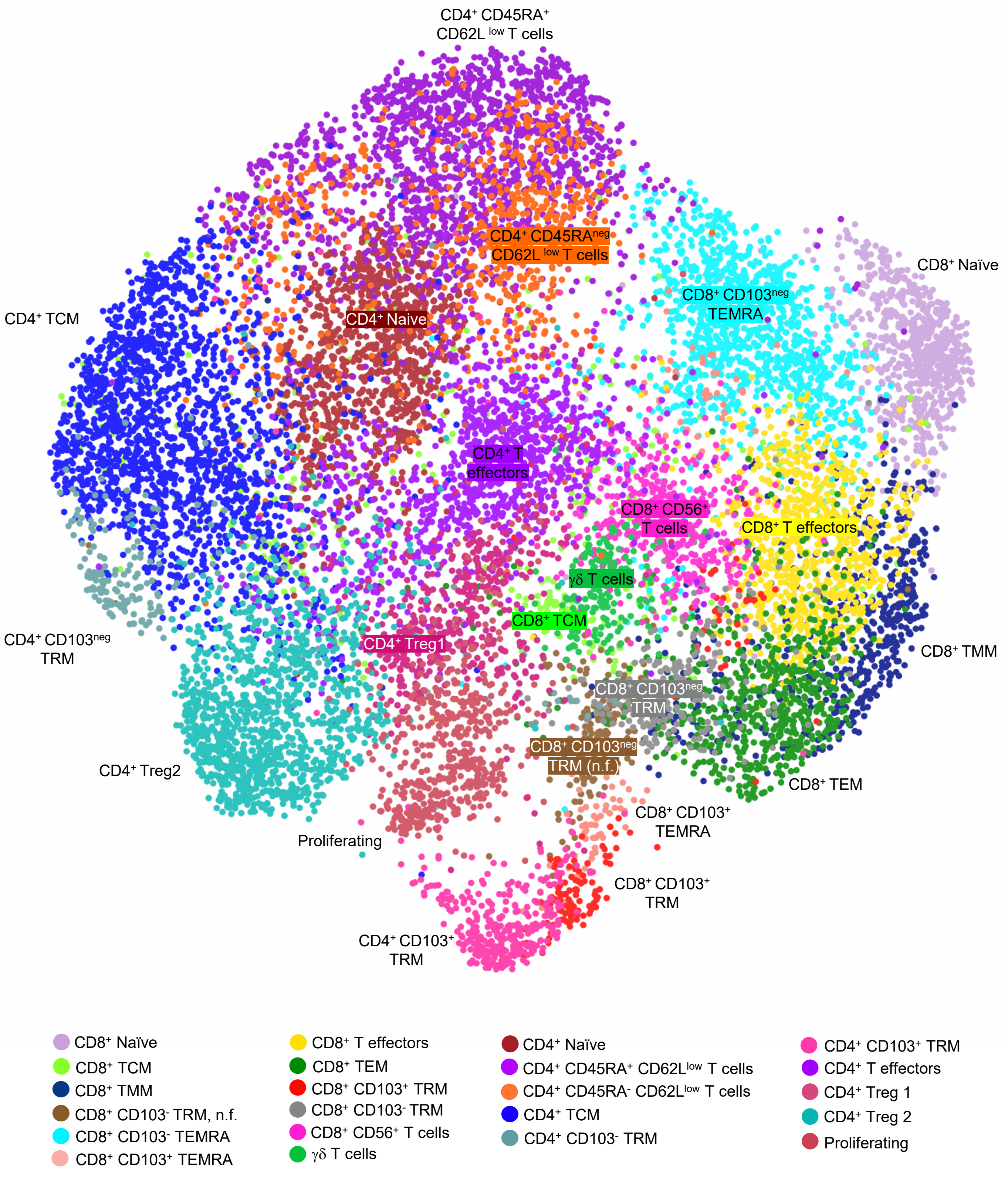

Supplemental Figure 3

Supplemental Figure 3. T cell clusters identified by scRNAseq + CITEseq of prospectively collected skin and blood samples from dtDHR and healthy control patients. UMAP showing 22 identified T cell clusters from integrating scRNAseq + CITEseq across 3 SJS/TEN, 3 MDE, 3 healthy control skin and 3 healthy control blood samples. 1 healthy control skin sample was excluded due to low number of cells. Healthy control blood and skin were not paired.

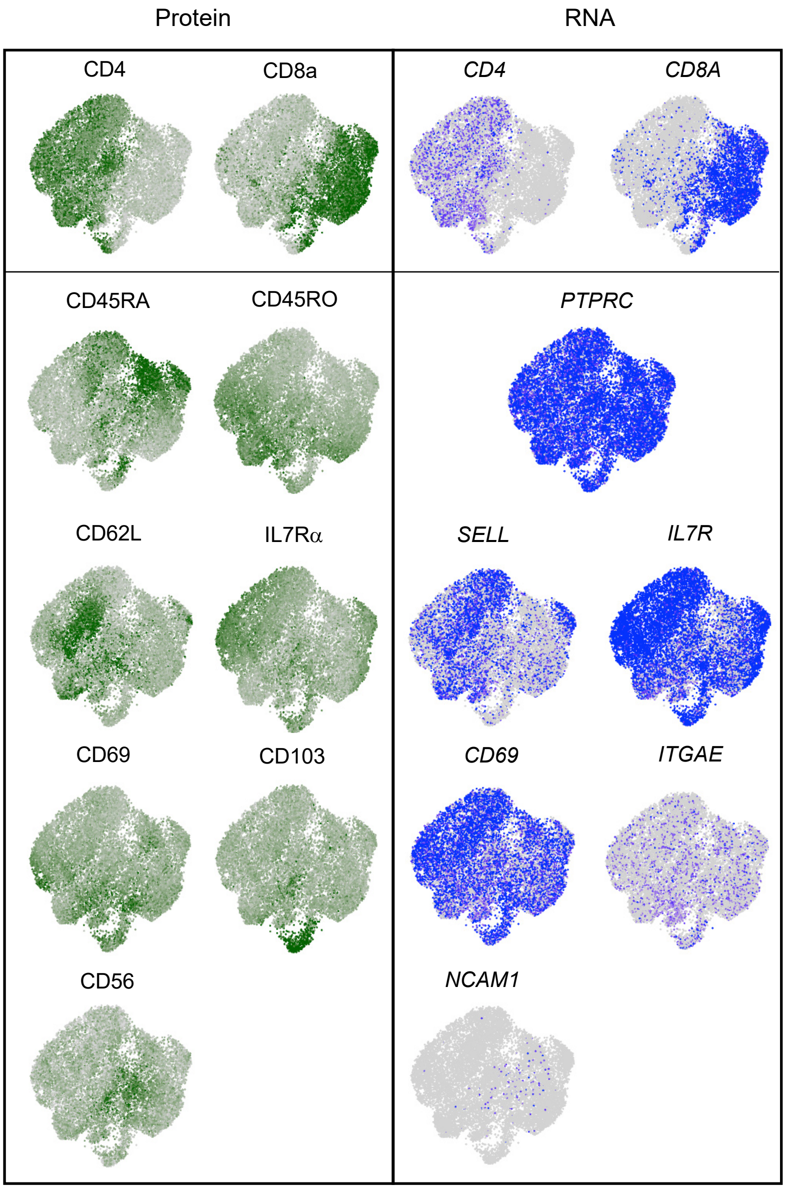

Supplemental Figure 4

Supplemental Figure 4. Comparison of key T cell phenotypic markers between CITEseq and scRNAseq. UMAPs of each key T cell phenotypic marker with improved resolution using protein (green) compared to RNA (blue). Distribution was largely comparable for CD4 and CD8 between the two modalities, though protein allowed for superior resolution so was used in defining each cluster. CITEseq allowed for discrimination between the isoforms of CD45RA and CD45RO that was not possible with scRNAseq alone. Cells positive for CD62L, IL7Rα, CD69, CD103 and CD56 were more clearly identifiable using CITEseq compared to scRNAseq alone.

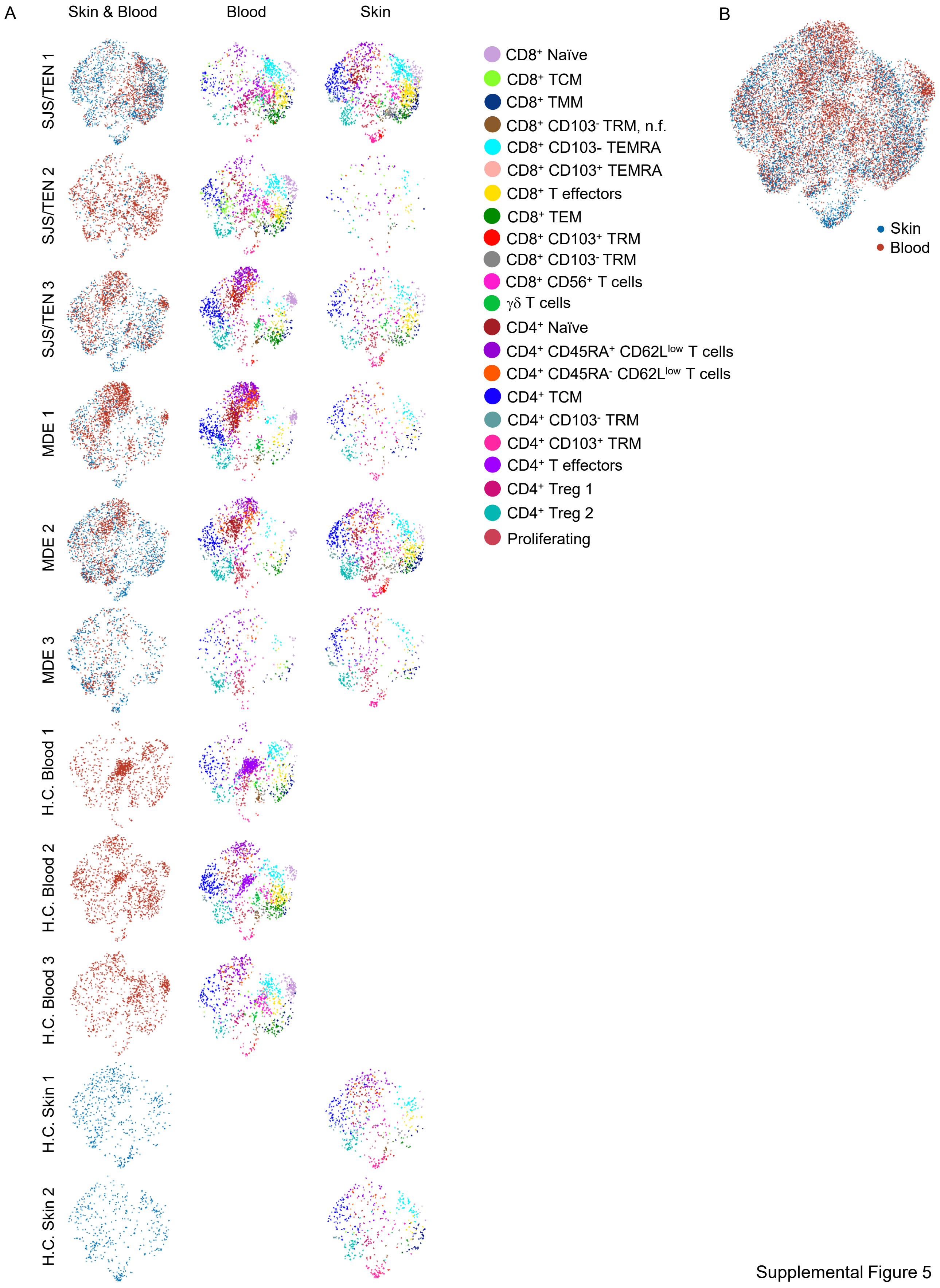

Supplemental Figure 5

Supplemental Figure 5. Distribution of cells in skin and blood of each patient using the joint (skin + blood) clustering analysis. **(A)** UMAPs showing combined skin (blue) and blood (red) per patient and healthy controls (H.C.) with comparative UMAPs showing skin and blood separately by cluster per patient. **(B)** UMAP showing all T cells from all samples by skin (blue) and blood (red).

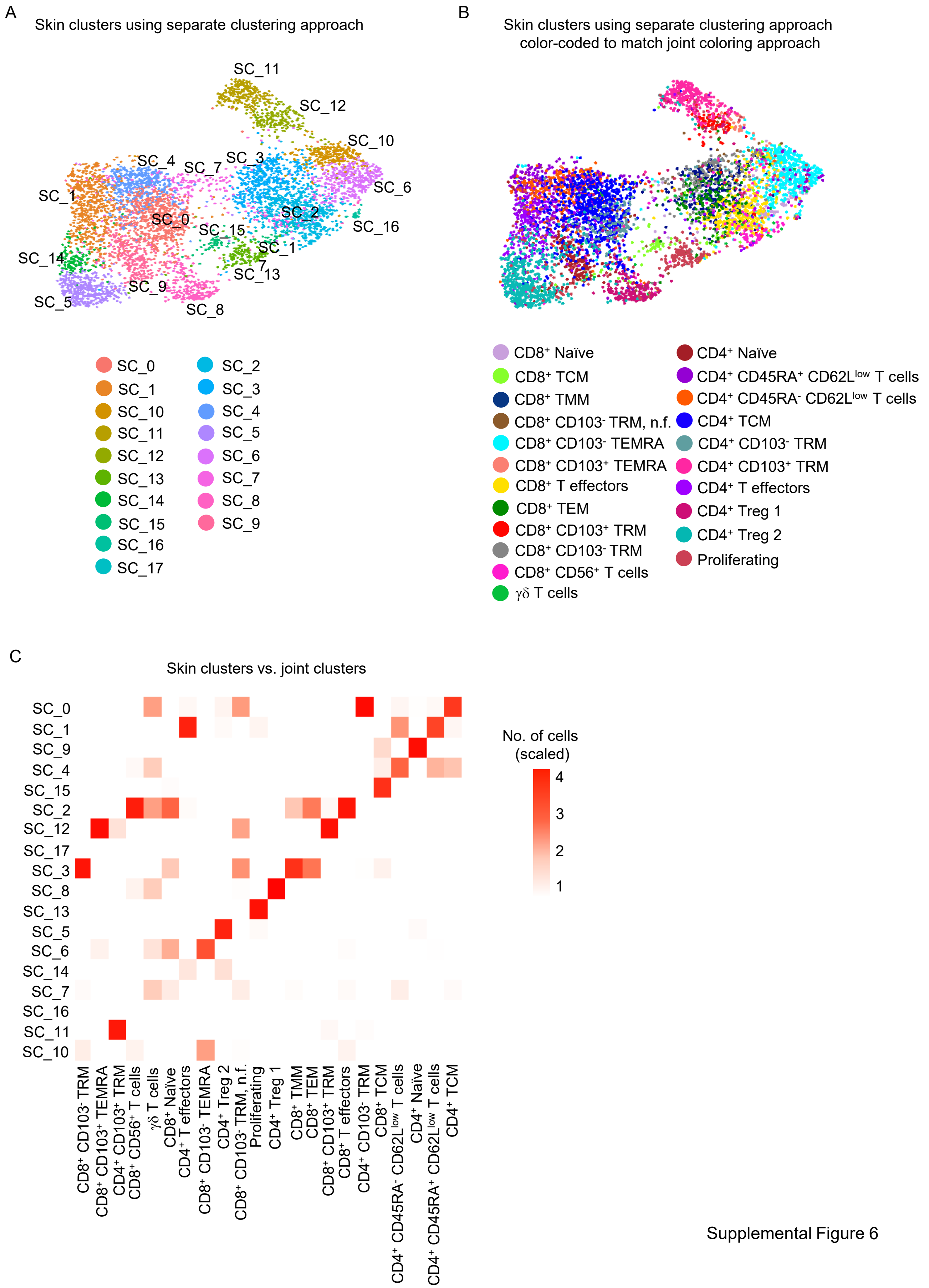

Supplemental Figure 6

Supplemental Figure 6. Skin-specific analysis mirrors joint clustering results. (A) UMAP showing skin-specific clustering (18 clusters) when skin is analyzed separately. **(B)** UMAP showing skin analyzed separately but now each cell is labelled using the annotations from the joint (skin + blood) clustering approach. **(C)** Heatmaps comparing clusters between joint (skin + blood) clustering approach and skin-specific clustering approach. Heatmaps show the number of cells that fall into each skin-specific cluster versus each joint cluster. The near diagonalization of both heatmaps indicates a considerable match between the joint clustering approach and tissue-specific clustering approach.

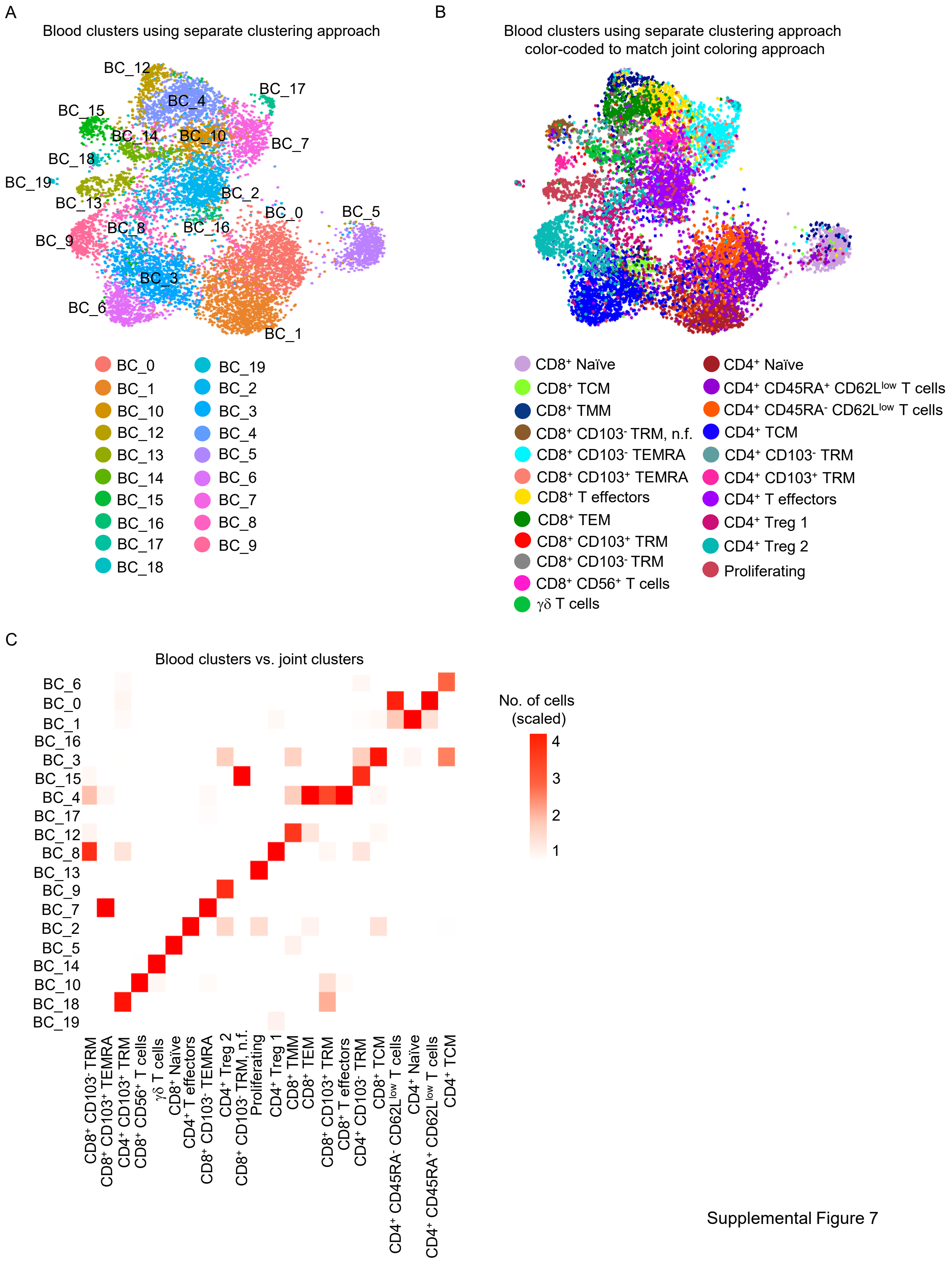

Supplemental Figure 7

Supplemental Figure 7. Blood-specific analysis mirrors joint clustering results. (A) UMAP showing blood-specific clustering (19 clusters) when blood is analyzed separately. **(B)** UMAP showing blood analyzed separately but now each cell is labelled using the annotations from the joint (skin + blood) clustering approach. **(C)** Heatmaps comparing clusters between joint (skin + blood) clustering approach and blood-specific clustering approach. Heatmaps show the number of cells that fall into each blood-specific cluster versus each joint cluster. The near diagonalization of both heatmaps indicates a considerable match between the joint clustering approach and tissue-specific clustering approach.

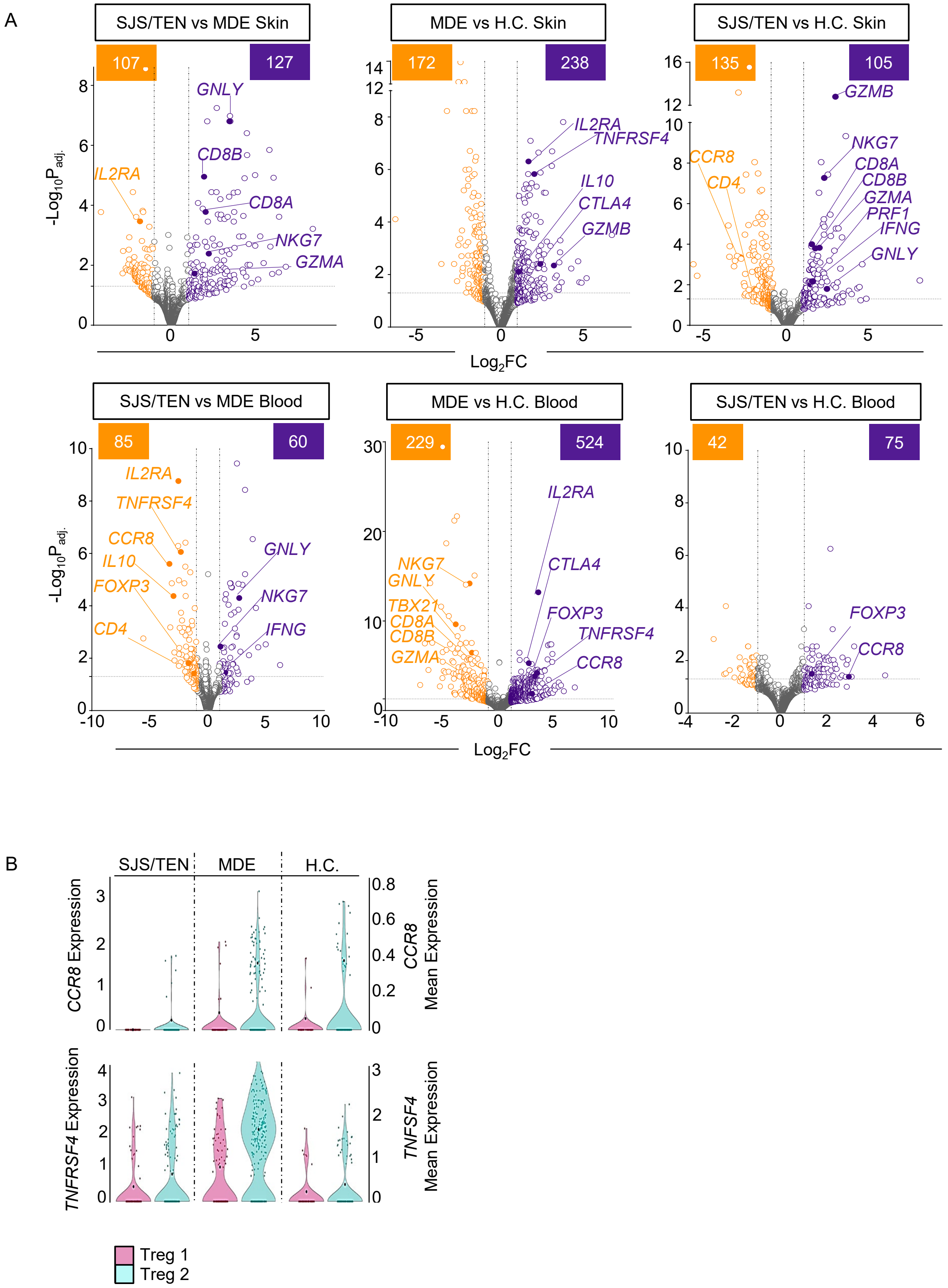

A
SJS/TEN vs MDE Skin
MDE vs H.C. Skin
SJS/TEN vs H.C. Skin
107
127
172
238
135
105
GNLY
CD8B
IL2RA
CD8A
NKG7
GZMA
IL2RA
TNFRSF4
IL10
CTLA4
GZMB
GZMB
CCR8
CD4
NKG7
CD8A
CD8B
GZMA
PRF1
IFNG
GNLY
-Log10Padj.
Log2FC
SJS/TEN vs MDE Blood
MDE vs H.C. Blood
SJS/TEN vs H.C. Blood
85
60
229
524
42
75
IL2RA
TNFRSF4
CCR8
IL10
FOXP3
CD4
GNLY
NKG7
IFNG
NKG7
GNLY
TBX21
CD8A
CD8B
GZMA
IL2RA
CTLA4
FOXP3
TNFRSF4
CCR8
FOXP3
CCR8
-Log10Padj.
Log2FC
B
SJS/TEN
MDE
H.C.
CCR8 Expression
CCR8 Mean Expression
TNFRSF4 Expression
TNFSF4 Mean Expression
Treg 1
Treg 2

Supplemental Figure 8. MDE displays a pro-Treg profile compared to SJS/TEN and healthy controls. (A) Volcano plots highlighting selected significantly differentially expressed genes between pooled SJS/TEN, MDE and healthy control (H.C.) skin and blood by pseudo-bulk analysis. Significance defined as Log2FC $\geq \pm 1$ and $P_{adj} < 0.05$, DESeq2, Wald test. **(B)** Violin plots showing expression (left y-axis) of *CCR8* and *TNFRSF4* in Treg clusters in SJS/TEN, MDE and H.C. skin. Violin plots show gene expression (left y-axis) and mean expression (right y-axis and visualized by black dot).

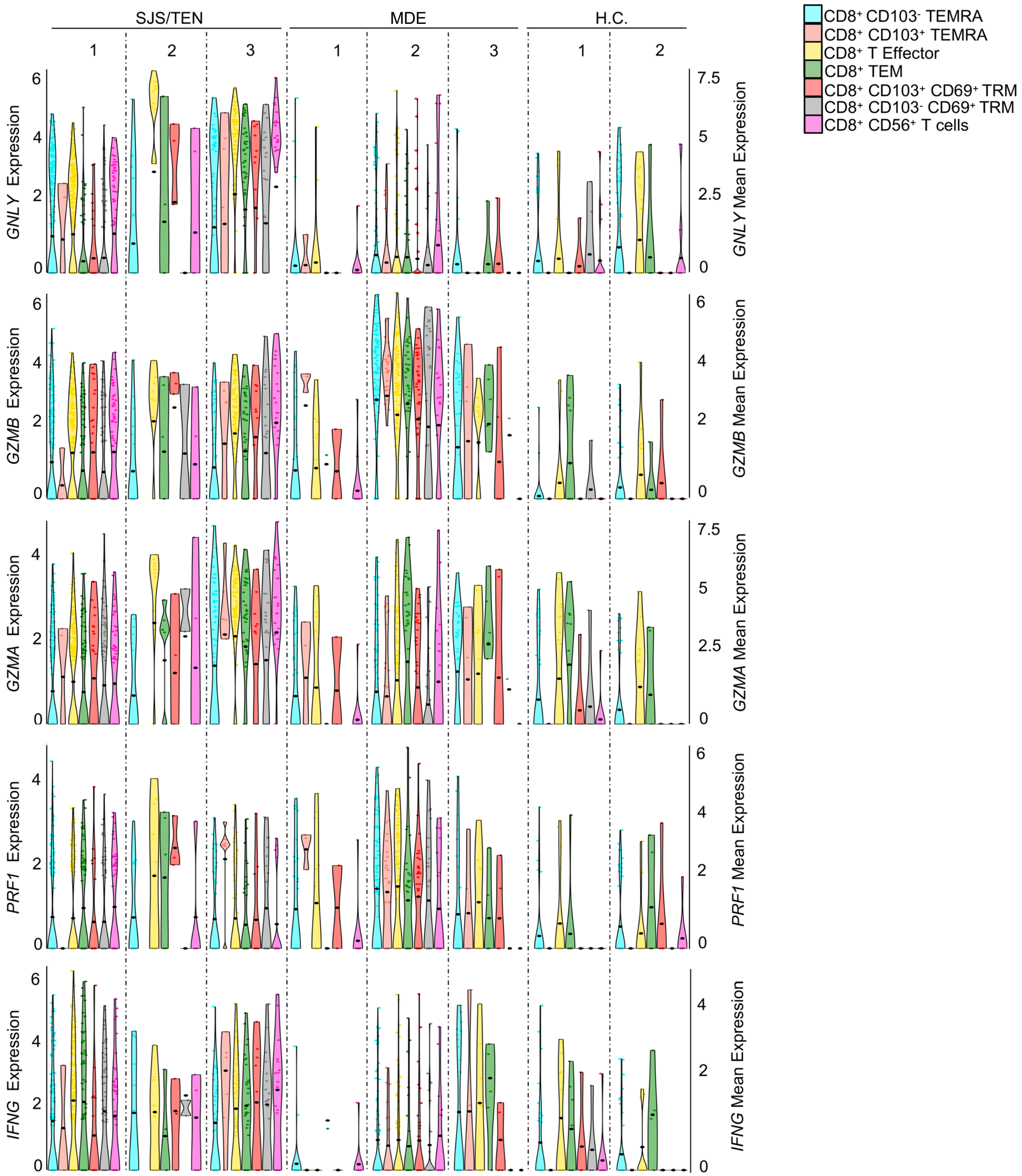

Supplemental Figure 9

Supplemental Figure 9. Expression of cytotoxic markers in skin at a single cell level per cytotoxic CD8$^+$ T cell cluster for each patient. Violin plots showing expression (left y-axis) of *GNLY*, *GZMB, GZMA, PRF1,* and *IFNG* in skin of each cytotoxic CD8$^+$ T cell cluster of each patient. Mean expression (right y-axis) is visualized with black dots.

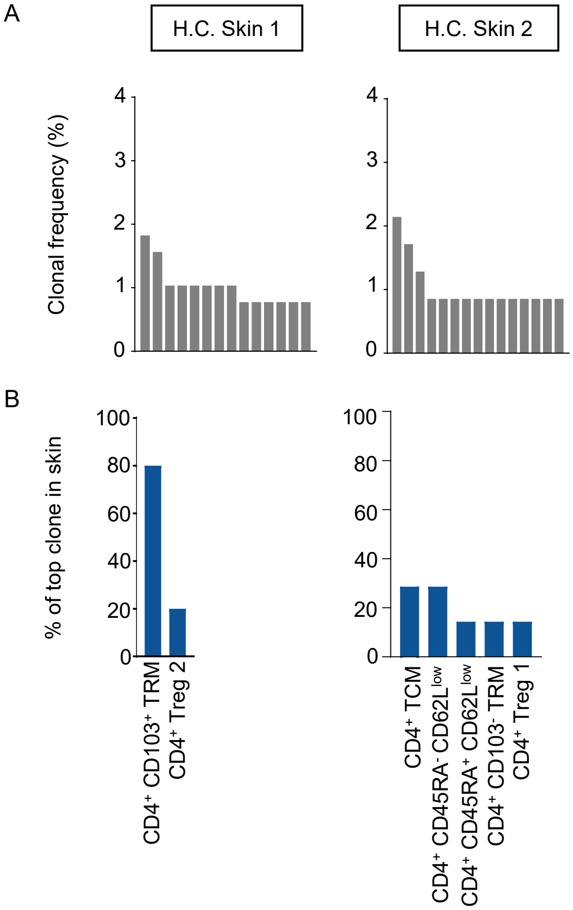

Supplemental Figure 10

Supplemental Figure 10. TCRseq analysis of healthy control skin. (A) Clonal frequency (percentage) of the top 15 clones in skin of each healthy control. **(B)** Bar graph showing percent distribution across T cell phenotypic clusters of the top clone in skin.

Lymphopenic MDE

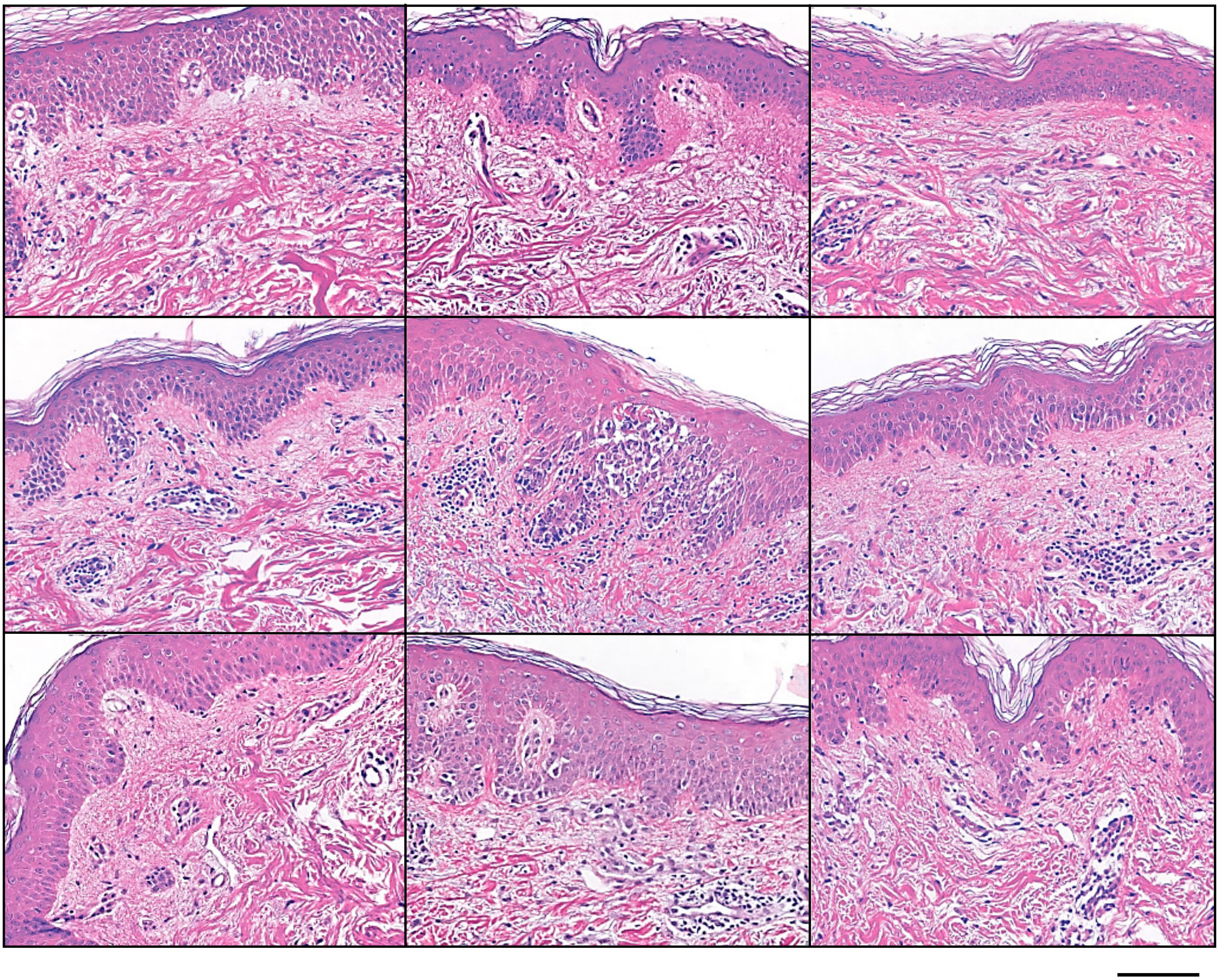

Supplemental Figure 11

Healthy

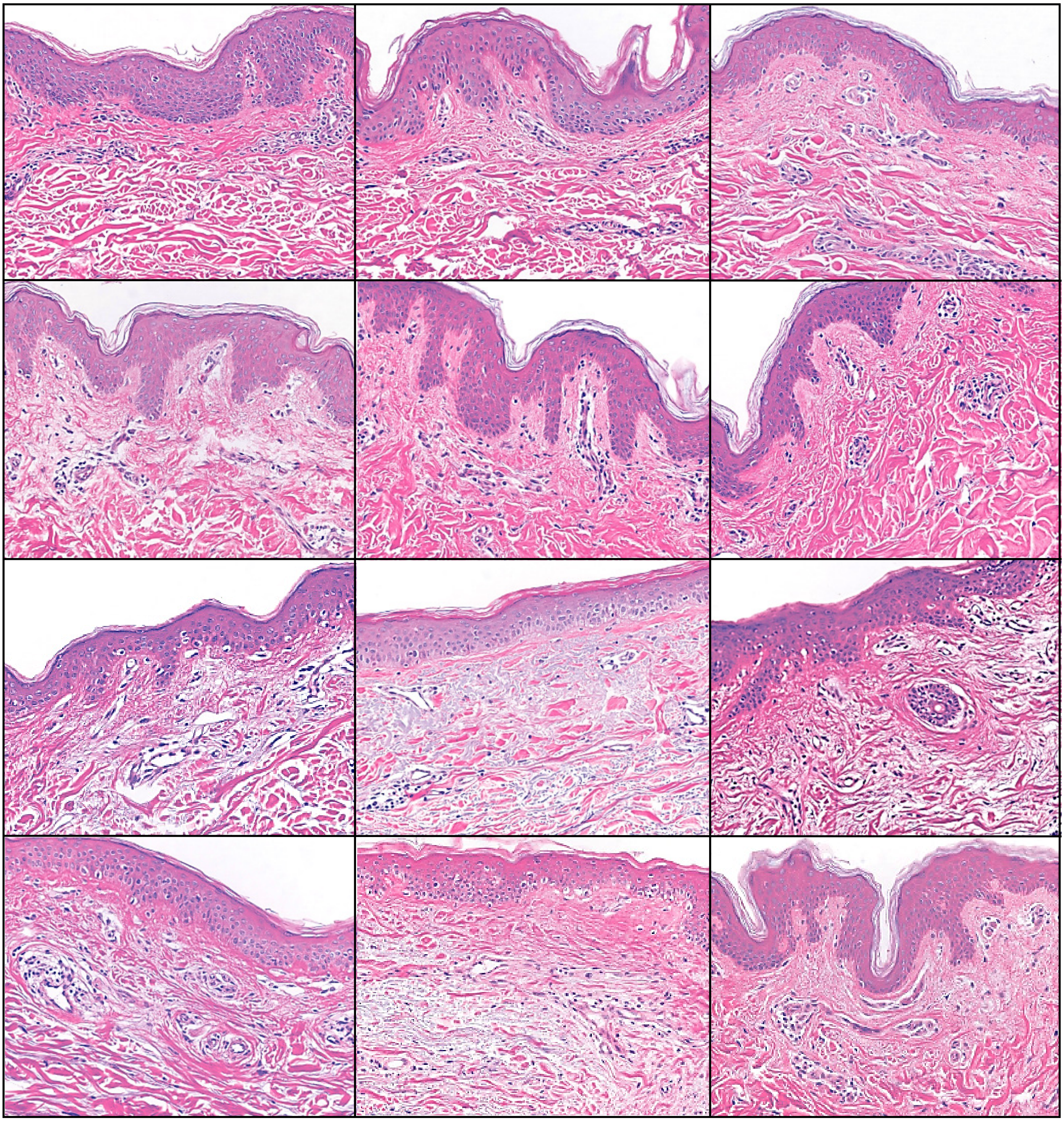

Supplemental Figure 11 continued

Supplemental Figure 11. Representative skin histology from lymphopenic MDE patients and healthy controls. Representative images of hematoxylin and eosin stained skin samples from lymphopenic MDE patients (N=9) and healthy controls (N=12) are shown. Black bar = 100 μm. Disclosure: Top left image of lymphopenic MDE and row 2 middle image of healthy were shown in Figure 5B.

A

B

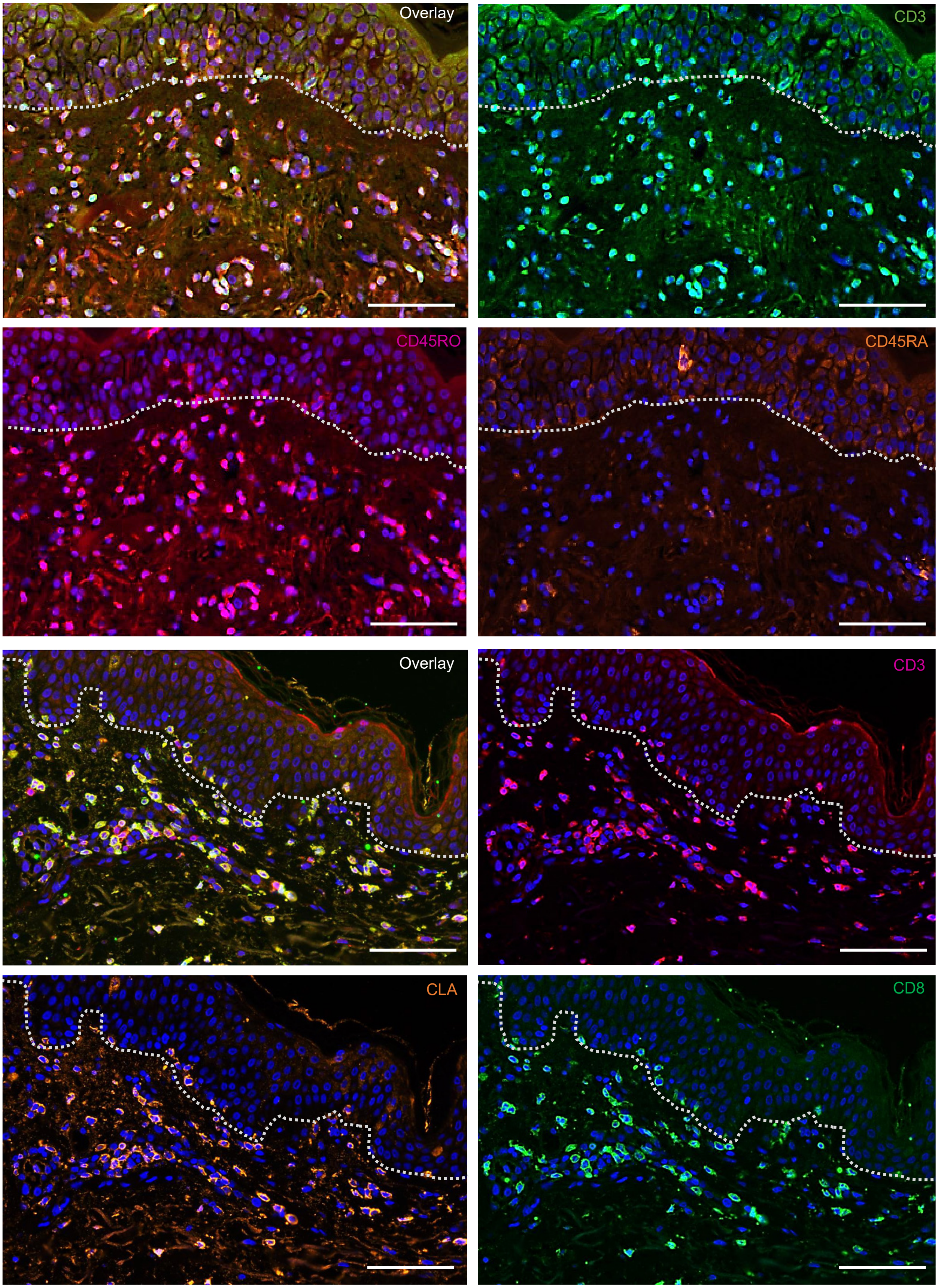

Supplemental Figure 12

Supplemental Figure 12. Characterization of T cell infiltrate in skin of lymphopenic MDE patients by immunofluorescence staining. (A) Representative images showing overlay, CD3 (green), CD45RO (magenta), CD45RA (orange) and DAPI nuclear stain (blue) in a lymphopenic MDE patient. **(B)** Representative images showing overlay, CD3 (magenta), CLA (orange), CD8 (green) and DAPI nuclear stain (blue) in a lymphopenic MDE patient. N=5 lymphopenic and healthy patients each stained. Gray dotted line depicts dermoepidermal junction. White lines = 100 μm.

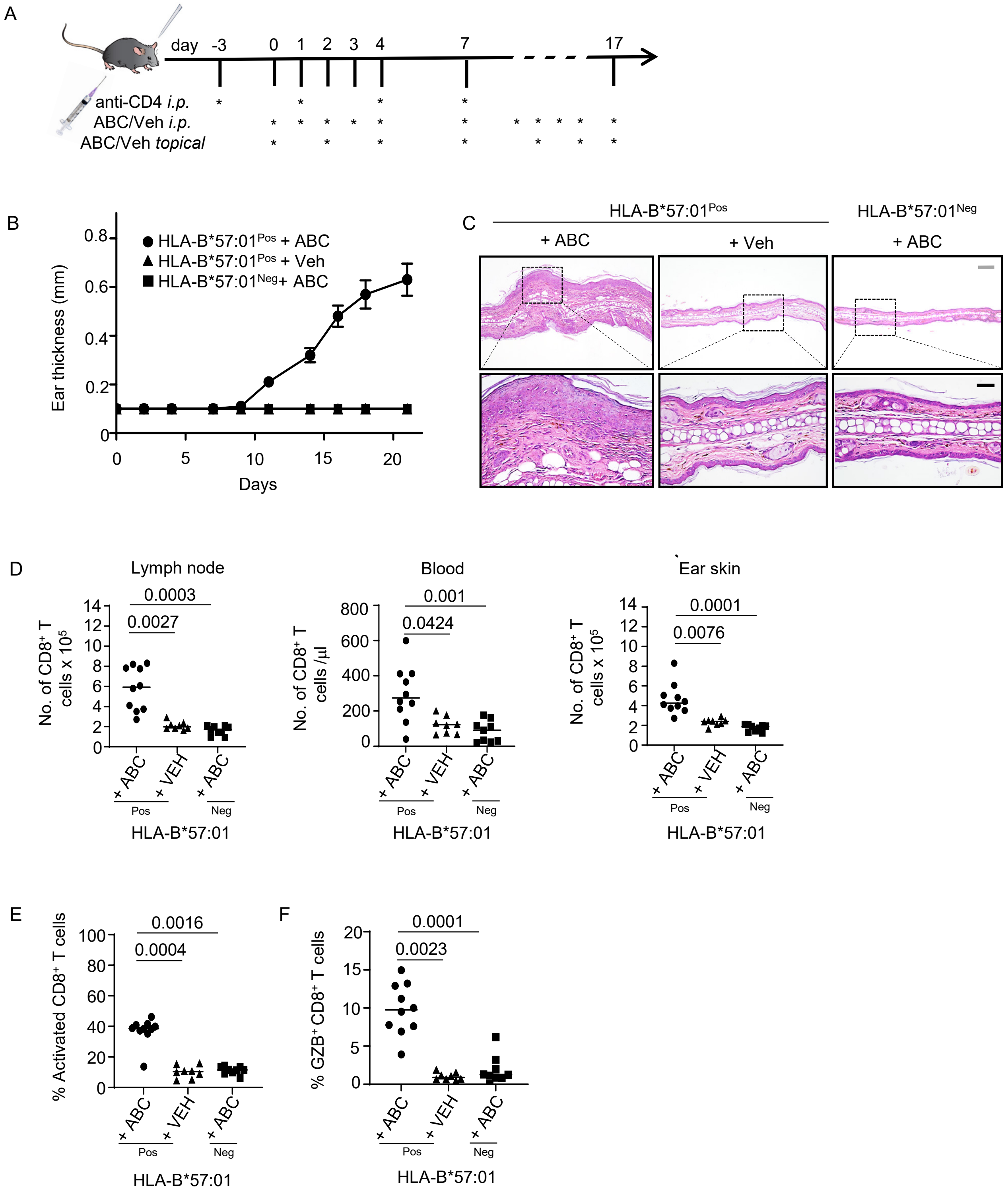
A
day -3 0 1 2 3 4 7 17
anti-CD4 i.p.
ABC/Veh i.p.
ABC/Veh topical
B
HLA-B*57:01Pos + ABC
HLA-B*57:01Pos + Veh
HLA-B*57:01Neg+ ABC
Ear thickness (mm)
Days
C
HLA-B*57:01Pos
HLA-B*57:01Neg
+ ABC
+ Veh
+ ABC
D
Lymph node
Blood
Ear skin
0.0003
0.0027
0.001
0.0424
0.0001
0.0076
No. of CD8+ T cells x 105
No. of CD8+ T cells /µl
+ ABC
+ VEH
+ ABC
Pos
Neg
HLA-B*57:01
E
F
0.0016
0.0004
0.0001
0.0023
% Activated CD8+ T cells
% GZB+ CD8+ T cells

Supplemental Figure 13. Abacavir induces $CD8^+$ T cell mediated dermatitis in HLA-B*57:01^{pos} mice.

(A) Schematic of the primary exposure mouse model. HLA-B*57:01^{pos} and HLA-B*57:01^{neg} mice were depleted of $CD4^+$ T cells and then treated topically to ear skin and systemically by i.p. injection with abacavir (ABC) or vehicle control (Veh) for 17 days. Mice developed ear dermatitis as determined by **(B)** ear thickness (mean with SEM) and **(C)** histology (gray bar = 200 µm, black bar = 50 µm). **(D)** Total number of $CD8^+$ T cells in cervical lymph nodes, blood, and ear skin by flow cytometry. **(E)** Percent activated (defined as $CD44^{high}CD62L^{low}CD69^+$) $CD8^+$ T cells in ear skin by flow cytometry. **(F)** Percent granzyme B (GZB) producing $CD8^+$ T cells in ear skin by flow cytometry. **(D-F)** Gated on $CD3^+CD8^+$ T cells. Lines show median. Significance defined as $P < 0.05$, Kruskal-Wallis test followed by Dunn's multiple comparisons test comparing HLA-B*57:01^{pos} mice receiving ABC to each control. **(A-F)** Pooled results from two independent experiments shown.

Blood

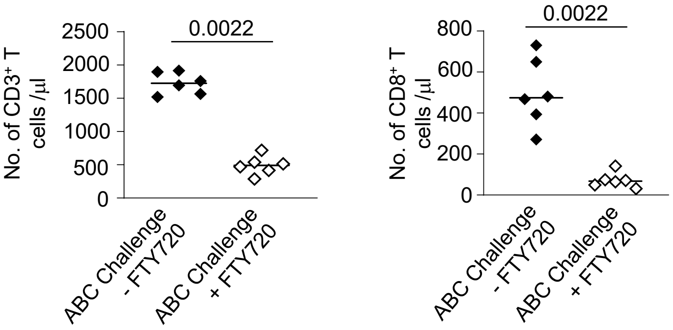

Supplemental Figure 14

Supplemental Figure 14. FTY720 depletes circulating T cells in mice.

Total number of $CD3^+$ T cells per µl and $CD8^+$ T cells per µl in blood post-challenge on day 107, with and without systemic treatment with FTY720 (assessed by flow cytometry). Lines show median. Significant, $P < 0.05$, two-tailed Mann-Whitney test. Pooled results from two independent experiments shown.

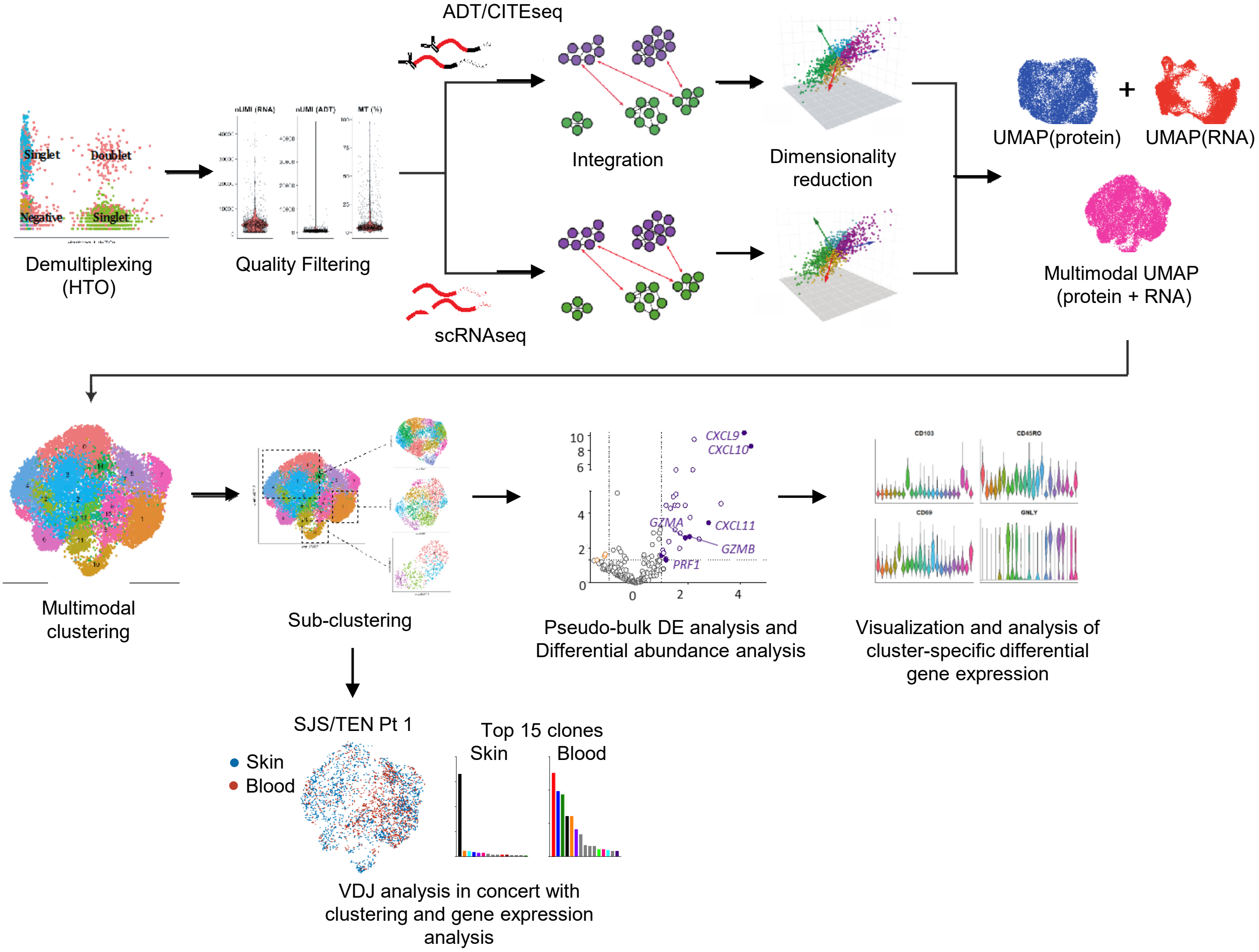

Supplemental Figure 15

Supplemental Figure 15. Schematic of scRNAseq + CITEseq analysis workflow. Data were demultiplexed using hashtag oligo (HTO) counts then both protein (ADT/CITE-seq) and RNA counts were quality filtered. Protein and RNA data were integrated then underwent dimensionality reduction. Individual and multimodal UMAPs were generated using Weighted Nearest Neighbor (WNN) method and compared to ensure proper integration, then clustering was performed on integrated data. From there, differential abundance analysis and pseudobulk differential expression analysis were performed, then cluster specific visualization and differential gene expression analysis was performed. In parallel, VDJ (TCRseq) analysis was performed in concert with clustering and gene expression analysis.

References

1 Watanabe, R. *et al.* Human skin is protected by four functionally and phenotypically discrete populations of resident and recirculating memory T cells. *Sci Transl Med* **7**, 279ra239, doi:10.1126/scitranslmed.3010302 (2015).
